# A High Temperature-Dependent Mitochondrial Lipase EXTRA GLUME1 Promotes Floral Phenotypic Robustness against Temperature Fluctuation in Rice (*Oryza sativa* L.)

**DOI:** 10.1371/journal.pgen.1006152

**Published:** 2016-07-01

**Authors:** Biyao Zhang, Shaohuan Wu, Yu’e Zhang, Ting Xu, Feifei Guo, Huashan Tang, Xiang Li, Pengfei Wang, Wenfeng Qian, Yongbiao Xue

**Affiliations:** 1 State Key Laboratory of Molecular Developmental Biology, Institute of Genetics and Developmental Biology, Chinese Academy of Sciences and National Center for Plant Gene Research, Beijing, the People’s Republic of China; 2 University of Chinese Academy of Sciences, Beijing, the People’s Republic of China; 3 State Key Laboratory of Plant Genomics, Institute of Genetics and Developmental Biology, Chinese Academy of Sciences, Beijing, the People’s Republic of China; 4 Beijing Institute of Genomics, Chinese Academy of Sciences, Beijing, the People’s Republic of China; 5 Collaborative Innovation Center for Genetics and Development, Fudan University, Shanghai, the People’s Republic of China; National University of Singapore and Temasek Life Sciences Laboratory, SINGAPORE

## Abstract

The sessile plants have evolved diverse intrinsic mechanisms to control their proper development under variable environments. In contrast to plastic vegetative development, reproductive traits like floral identity often show phenotypic robustness against environmental variations. However, it remains obscure about the molecular basis of this phenotypic robustness. In this study, we found that *eg1* (*extra glume1*) mutants of rice (*Oryza savita* L.) showed floral phenotypic variations in different growth locations resulting in a breakdown of floral identity robustness. Physiological and biochemical analyses showed that *EG1* encodes a predominantly mitochondria-localized functional lipase and functions in a high temperature-dependent manner. Furthermore, we found that numerous environmentally responsive genes including many floral identity genes are transcriptionally repressed in *eg1* mutants and *OsMADS1*, *OsMADS6* and *OsG1* genetically act downstream of *EG1* to maintain floral robustness. Collectively, our results demonstrate that *EG1* promotes floral robustness against temperature fluctuation by safeguarding the expression of floral identify genes through a high temperature-dependent mitochondrial lipid pathway and uncovers a novel mechanistic insight into floral developmental control.

## Author Summary

Various mechanisms have evolved to ensure proper organ formation under variable environments in order to complete one organism’s life cycle. In angiosperms, vegetative and reproductive organs show a differential plastic development between varied environments, with a low plasticity or high robustness for flower formation, but little is known about its intrinsic mechanism. Here we report that gene *EG1* (*EXTRA GLUME1*) can enhance the floral robustness against temperature fluctuation in rice. *EG1* encodes a predominantly mitochondria-localized functional lipase and its loss of function disrupts floral development in a high temperature-dependent manner. In consistent, both *EG1* and its lipase activity are positively induced by high temperature. Transcriptomic and genetic analyses revealed that *EG1* functions upstream of several floral identity genes, eg, *OsMADS1*, *OsMADS6* and *OsG1*. Taken together, our results uncover a novel mitochondria-mediated lipid metabolic pathway to promote floral developmental robustness. Our findings may help to genetically improve floral traits of rice to maintain a stable yield when planted in different locations and/or under heat stress conditions.

## Introduction

The sessile plants have evolved various exquisite adaptive strategies to cope with environmental changes [1,2]. Among them, phenotypic plasticity is the ability of a single genotype capable of producing different phenotypes in response to varying environments [3–6]. For an integral high fitness, morphologies of vegetative organs of a single plant, such as roots, leaves and stems, require a high phenotypic plasticity [7–10], whereas that of reproductive organs, such as flowers, fruits and seeds, are always associated with low plasticity also known as phenotypic robustness/stability [11–15]. Thus, plants must coordinate the developments of these organs.

Compared with progresses in understanding the molecular mechanisms of high phenotypic plasticity [10,16–19], very little is known about the molecular basis of phenotypic robustness [20,21]. Recent studies have shown that there are a group of specific genes regulating the degree of phenotypic plasticity and determining the reaction norm of a trait among various environments, which are termed plasticity genes [22–24]. However, most of the identified plasticity genes are high plasticity-associated [16,25–27], only few promote phenotypic robustness [28–30]. Members of *HSP90* (*HEAT SHOCK PROTEIN 90*) family, as central hubs of numerous biological pathways, are required for maintenance of phenotypic robustness in both animals and plants [28,31–34]. MSH1 (MutS HOMOLOG1), a homolog of bacterial mismatch repair protein MutS, has been reported to repress the developmental plasticity of plant architecture, leaf morphology and flowering time in several dicot and monocot plants [29,35]. A nuclear protein RPL1 (RICE PLASTICITY1) in rice also appears to promote the relatively stable plant architecture and panicle structure between different environments [30]. Despite these discoveries, we still know very little about the molecular mechanisms of phenotypic robustness, especially that of plant reproductive traits. In addition to the known epigenetics-dependent transcriptional regulation and hormone signaling [20,29–31,35], lipid homeostasis is also known to influence phenotypic robustness [36,37]. Coordinated regulations of cellular lipid homeostasis are crucial to organisms’ adaptive robustness under severe temperatures [37–40]. Furthermore, lipid-related synthetases and lipases can also be regulated at transcriptional and posttranslational levels to influence lipid homeostasis [41,42]. Among them, mitochondria-associated lipid metabolism is key to the lipid homeostasis [43]. For instance, Arabidopsis seedlings with decreased cardiolipin in mitochondrial membrane are easier to turn yellow and necrotic under extended darkness or heat due to a failure of mitochondrial morphogenesis, showing a lowered stability [41,44,45]. However, it remains unclear whether mitochondria also mediate the phenotypic robustness in plant reproductive organs.

Flower morphology, as a gold standard in plant taxonomy, has the most remarkable robustness within and between individuals of the same population [11,46], making it an ideal trait for studies on the molecular basis of phenotypic robustness against environmental fluctuation. Nevertheless, so far no gene has been identified to regulate the phenotypic robustness of floral identity, although several environment-dependent floral mutants have been reported [47–52]. We previously found that a rice floral mutant *eg1* (*extra glume1*) exhibited a floral variation in different growth conditions [53], implying that *EG1* is likely involved in floral robustness. Recent studies have shown that *EG1* encodes a putative lipase regulating rice floral identity and meristem determinacy [53]. It also functions in JA (jasmonic acid) biosynthesis to promote the expression of floral identity gene *OsMADS1* through an *EG2*/*OsJAZ1*, *OsCOIb* and *OsMYC2-* mediated JA signaling pathway [54], similar to its homologous genes *AtDAD1* (*DEFECTIVE IN ANTHER DEHISCENCE1*) and *AtDGL* (*DONGLE*) in Arabidopsis [55,56]. In this study, we find that *EG1* is a predominantly mitochondria-localized functional lipase and promotes floral robustness against temperature fluctuation in a high temperature-dependent manner. Collectively, our results reveal a novel molecular mechanism underlying floral phenotypic robustness.

## Results

### *eg1* shows high plasticity in floral identity through an interaction of genotype and environment

**Fig 1:**
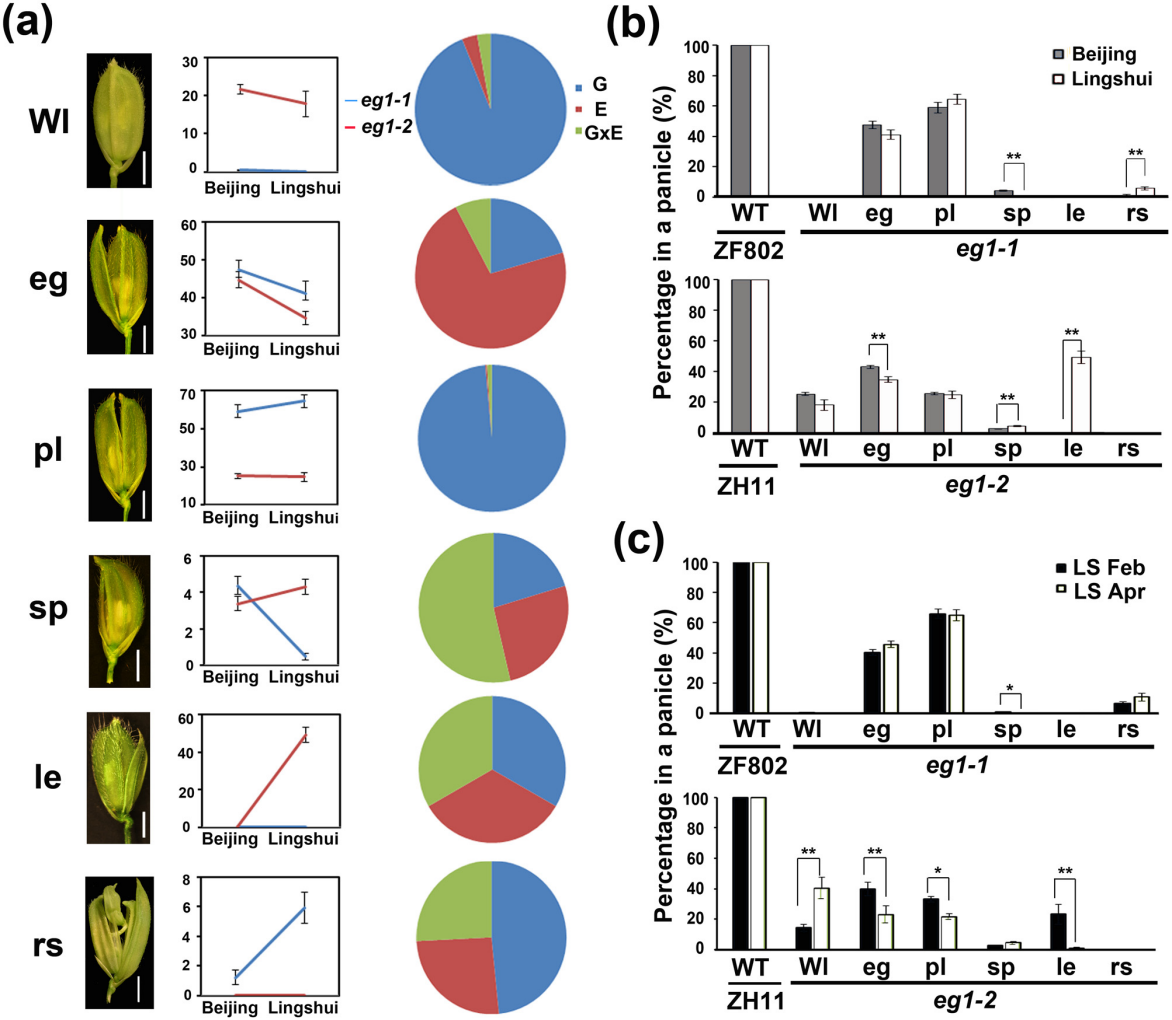
Phenotypic plasticity of floral identities of *eg1*. (a) Morphology and plasticity analysis of six variable phenotypes of *eg1* spikelets. Floral structures and two-factor plots with percentages per panicle in the y axis of all variable phenotypes are shown in the left and middle columns, respectively. Effects of G, E and GxE on each variable phenotype shown in the pie charts (right column) were analyzed by two-way ANOVA. Bar = 2 mm in floral structures. (b) Statistical analysis of the phenotypic plasticity of *eg1* and wild-type spikelets in two different planting locations. Beijing, Beijing summer. Lingshui, Lingshui winter. (c) Statistical analysis of the phenotypic plasticity of *eg1* and wild-type spikelet in two different planting seasons. LS Feb, Lingshui Feb. LS Apr, Lingshui Apr. Wl, WT-like; eg, extra glume; pl, palea to lemma; sp, smaller pa; le, long empty glumes; rs, reiterated spikelets. Values are means ± SE, number of analyzed panicles ≥ 20 in (b), ≥ 10 in (c). Significant difference was determined by ANOVA, *P < 0.05, **P < 0.01.

Previously, we found that *eg1* displayed a floral identity variation possibly influenced by growth conditions [53]. To examine if this variability was mainly due to the environmental alterations, we analyzed the spikelet phenotypes of *eg1-1* (in *indica* ZF802 background) and *eg1-2* (in *japonica* ZH11 background) in two groups of separate environments (Fig 1) and found that the floral phenotypic variability of *eg1* is likely caused by both genotype and environment. To define the phenotypic variability, we divided the spikelet phenotypes of *eg1* into six groups, which were called variable phenotypes, including Wl (WT-like), eg (extra glume), pl (palea to lemma), sp (smaller pa), le (long empty glumes) and rs (reiterated spikelets) (Fig 1A, S1 Fig and S1 Table). The results showed that sp and rs of *eg1-1* as well as Wl, le, eg and sp of *eg1-2* exhibited significant plasticity between two environments, especially le of *eg1-2*, which displayed the highest plasticity (Fig 1B and 1C), suggesting that environment also contributes to the variations of floral phenotypes of *eg1*. To further examine the relationships of genotype (G), environment (E), genotype-environment interaction (GxE) and the variable phenotypes, we calculated their effects on phenotypes by two-factor ANOVA and found that Wl and pl were affected mainly by G, eg by both E and G but rarely by GxE, sp, le and rs by all three factors, and among them, le could serve as a marker for the phenotypic plasticity of *eg1-2* due to its large proportion in a panicle and opposite phenotypes between two environments (Fig 1A). Taken together, these results showed that *eg1* shows higher floral plasticity, suggesting that *EG1* promotes the floral robustness in rice.

### Allelic *eg1* and their genetic backgrounds together regulate the *eg1* floral plasticity

**Fig 2:**
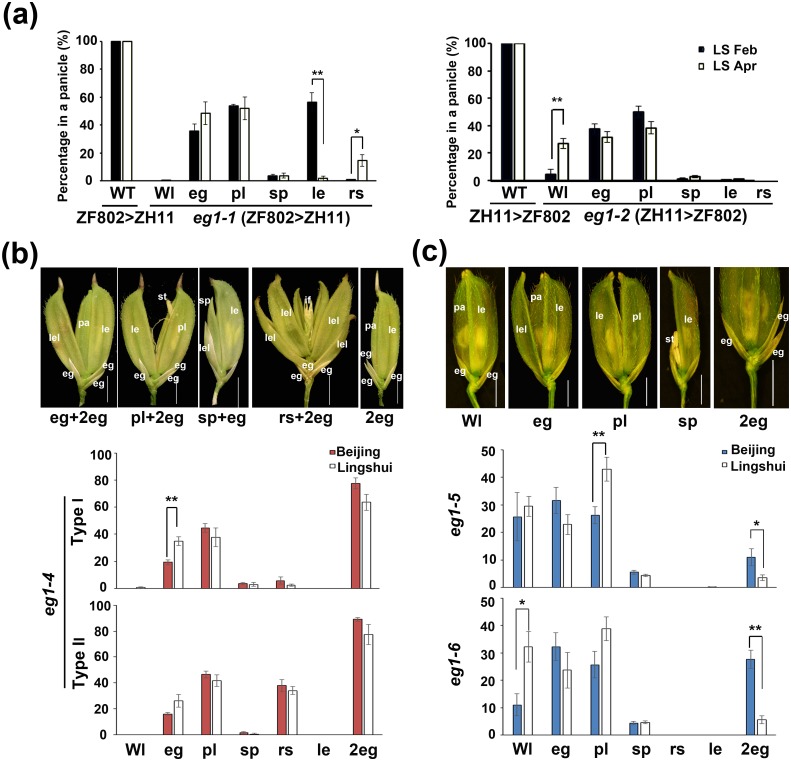
Subspecific variations of *eg1* floral plasticity. (a) Floral plasticity of *eg1* alleles in two exchanged backgrounds. *eg1-1* (ZF802>ZH11) and *eg1-2* (ZH11>ZF802) show *eg1-1* and *eg1-2* backcrossed into ZH11 or ZF802 backgrounds, respectively. (b) Floral plasticity of *eg1-4* in *indica* Dular background. Statistical analysis of two types of panicles according to rs (Type I and Type II) are shown. (c) Floral plasticity of *eg1-5* and *-6* in *japonica* Nipponbare background. Statistical analysis of the two independent lines are shown. LS Feb, Lingshui Feb. LS Apr, Lingshui Apr. Beijing, Beijing summer. Lingshui, Lingshui winter. Variable phenotypes of spikelets are defined as in S1 Table, and percentages of them in a panicle are shown in the y axis. le, lemma; pa, palea; st, stamen; eg, empty glume; if, inflorescence primordia; sp, smaller pa; lel, lemma-like organ; pl, palea-lemma mosaic organ. Bars = 2 mm. Values are means ± SE, number of analyzed panicles ≥ 5, and significant difference was determined by ANOVA, *P < 0.05, **P < 0.01.

To further examine the influence of genotype on the floral plasticity of *eg1*, we swapped the genetic backgrounds of two *eg1* alleles. *eg1-1* in a largely *japonica* background showed high phenotypic plasticity especially for le and rs phenotypes, similar to *eg1-2* (ZH11), whereas *eg1-2* in an *indica*-dominant background showed low floral plasticity similar to *eg1-1* (ZF802) (Fig 2A), indicating that genetic backgrounds also influence the phenotypic plasticity of *eg1* spikelets.

To verify this finding, we further used CRISPR/Cas9 technology to construct *eg1* alleles in Nipponbare (*japonica*) and Dular (*indica*) backgrounds. Two types of spikelet phenotypes were found in *eg1-4* allele with Dular background and both showed low plasticity (Fig 2B), while *eg1-5* and *-6* alleles in Nipponbare background showed relatively higher plasticity than alleles in two *indica* backgrounds ZF802 and Dular, similar to that in *japonica* ZH11 (Fig 2C and S2 Fig), suggesting that the floral plasticity of *eg1* alleles in *japonica* backgrounds tend to be higher than that in *indica* backgrounds. In another aspect, *eg1* alleles in *indica* backgrounds had severer floral disturbance than that in *japonica* concerning Wl and rs phenotypes (Fig 2 and S1 Fig), suggesting that *EG1* has functions in both floral robustness and identity, which are differentiated in two subspecies. To explore the possible causes of these differentiation, we compared the *cis*-elements and expressional level of *EG1* in several *japonica* and *indica* varieties and discovered the correlative differences in both cis-elements and expressional levels between *japonica* and *indica* (two types) varieties (S3 Fig and S2 Table), which implied that transcriptional differences may be a crucial cause of functional differentiation of *EG1* in subspecies. All these results indicated that both *eg1* allelic variations and their genetic backgrounds regulate the floral plasticity of *eg1*.

### Temperature is a major environmental factor mediating the plastic development of *eg1* spikelets

**Fig 3:**
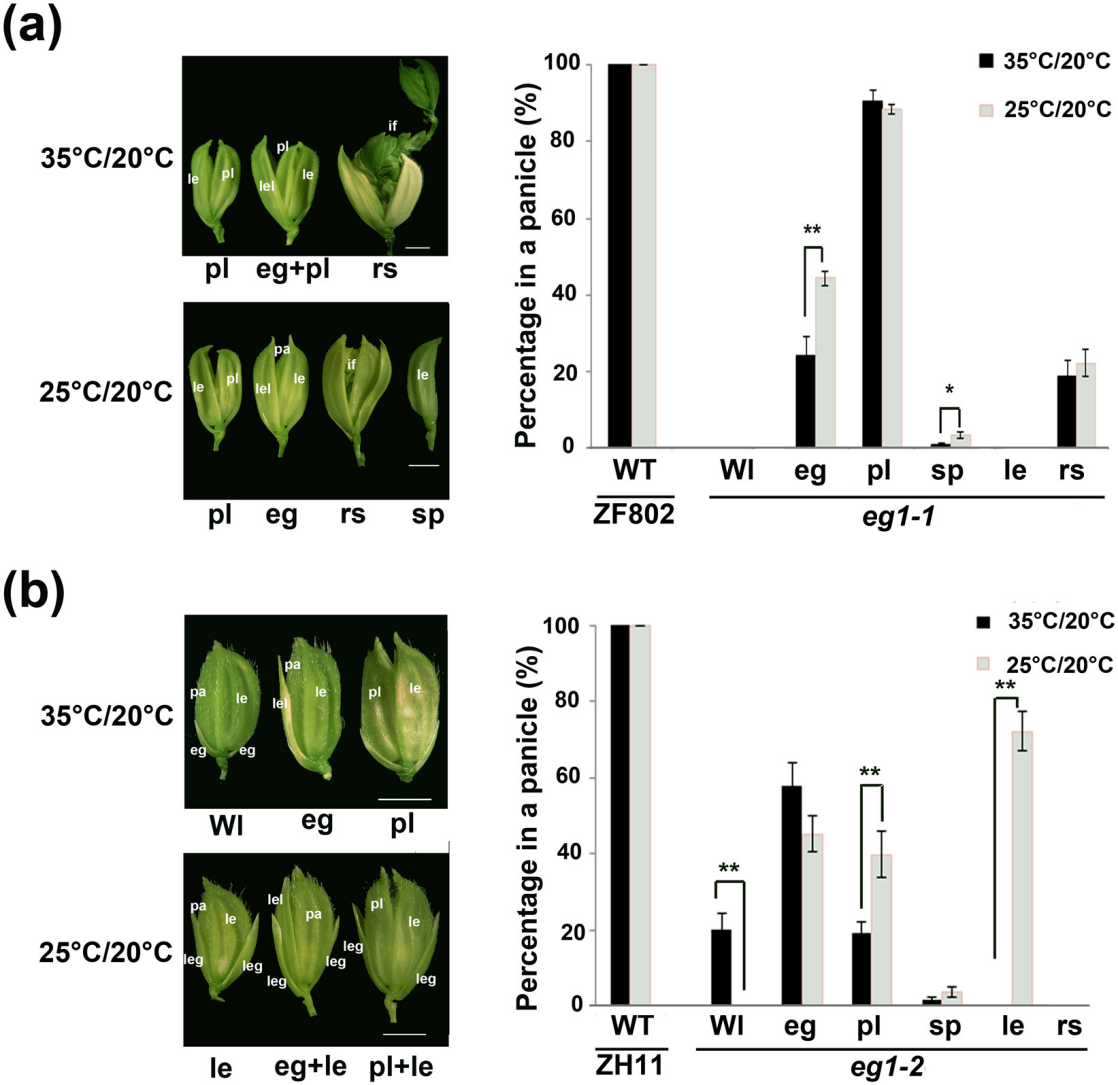
Temperature-dependent floral plasticity of *eg1*. (a) Floral plasticity of *eg1-1* (ZF802) under two different temperature conditions. (b) Floral plasticity of *eg1-2* (ZH11) under two different temperature conditions. Spikelet phenotypes and statistical analysis are shown in the left and right of (a) and (b). Variable phenotypes of spikelets are defined as in S1 Table. le, lemma; pa, palea; eg, empty glume; if, inflorescence primordia; lel, lemma-like organ; pl, palea-lemma mosaic organ; leg, long empty glume in spikelet structures. Bars = 2 mm. Values are means ± SE, number of analyzed panicles >10, and significant difference was determined by ANOVA, *P < 0.05, **P < 0.01.

In order to find out the environmental factors mediating the plastic development of *eg1* spikelets, we first compared the growing conditions for phenotypic analysis and found a marked difference in daily high temperatures of two environments (S4 Fig), suggesting that the temperature variation between two environments could be a major environmental factor influencing the *eg1* plasticity. To verify this prediction, floral plasticity of wild-types and *eg1* alleles were examined in two artificial growth chambers with 35°C light 12 hr / 20°C dark 12 hr and 25°C light 12 hr / 20°C dark 12 hr respectively, while other growth conditions were kept identical. The low plasticity of *eg1-1* and nearly 70% le phenotypes of *eg1-2* showed that the floral plasticity of *eg1* in the chambers was similar to and even higher than that under natural growth conditions (Fig 3). These results showed that temperature is a major environmental factor mediating the floral plasticity of *eg1*.

### *EG1* encodes a predominately mitochondria-localized functional lipase

**Fig 4:**
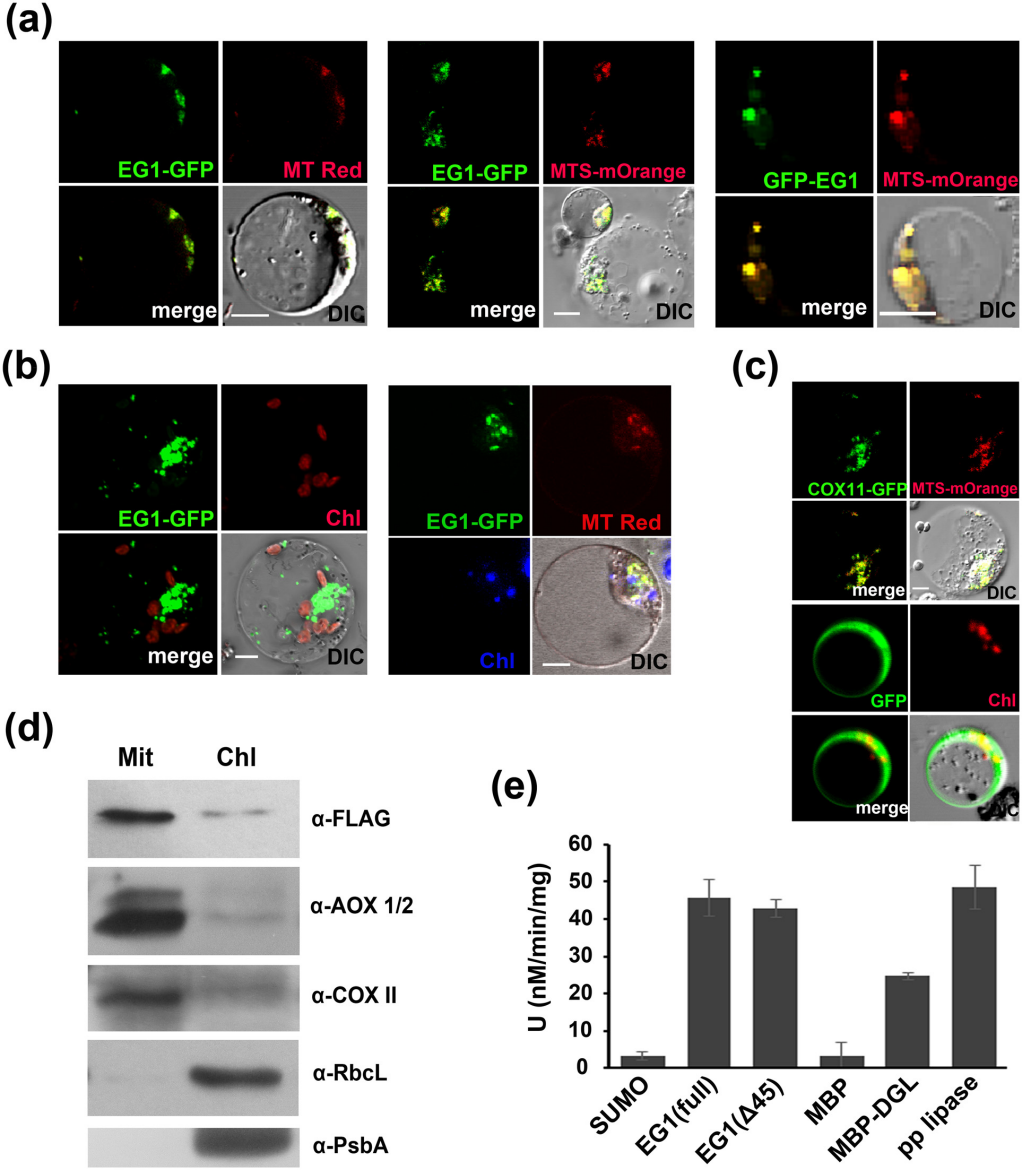
*EG1* encodes a functional lipase predominately localized in mitochondria. (a) Co-localization of EG1-GFP or GFP-EG1 fusion protein with mitochondria in rice protoplasts. Mitochondria are marked by dye Mito Tracker Red (MT Red) or MTS-mOrange protein. (b) Localization analysis of EG1-GFP and chloroplasts in rice protoplasts. An *EG1-GFP* driven by *35S* or native promoter is shown in the left and right, respectively. Chloroplasts are detected by its auto-fluorescence. Mitochondria are marked by Mito Tracker Red (MT Red). (c) Localization of mitochondrial (*35SPro*:*COX11-GFP*) [60] (up) and cellular (*35SPro*:*GFP*) (bottom) controls of rice protoplasts. DIC, pictures photographed by differential interference contrast microscope. Bar = 10 μm. (d) Subcellular fractionation assay. Mit, mitochondria fraction; Chl, chloroplasts fraction; α-FLAG, antibody of FLAG-EG1; α-AOX1/2 and α-COXII, specific antibodies of mitochondrial proteins AOX1/2 and COXII; α-RbcL and α-PsbA, specific antibodies of chloroplast proteins RbcL and PsbA. (e) Lipase activity of EG1 in vitro with P-nPB as a substrate at 30°C. EG1 (Full) and EG1 (Δ45) respectively refer to fusion proteins of full-length or no N-terminal (45 aa) protein of EG1 and SUMO peptide. DGL and pp lipase (porcine pancreatic lipase) were used as positive controls. Values are means ± SE for three independent experiments.

Previously, EG1 was shown to be localized in chloroplasts in transient expression assays [54]. However, EG1-like lipases appear to have variable subcellular locations [56–59]. To examine the subcellular localization of EG1 *in vivo*, two different EG1 and GFP fusion proteins driven by *35S* promoter were first expressed in rice protoplasts and were found to be co-localized with both mitochondrial specific dye Mito Tracker Red and mitochondrial maker protein MTS-mOrange [60] but hardly with chloroplast auto-fluorescence, and an EG1-GFP fusion protein driven by native promoter was also detected in mitochondria (Fig 4A and 4B), suggesting that EG1 protein is mainly, if not all, localized in mitochondria. To compare this finding with the previous one, the reported vector *pCAMBIA1301-Pro35S*:*EG1-GFP* [54] was also examined in our transient system and a similar localization was detected (S5 Fig). To further verify the EG1 localization, subcellular fractionations of one-week seedlings of *eg1-2* complementation lines with FLAG-EG1 (S6 Fig) were successively carried out and the EG1 fusion protein was predominately co-fractionated with mitochondria (Fig 4D), confirming the mitochondrial localization of EG1 *in vivo*. Taken together, these results showed that *EG1* encodes a protein predominately localized in mitochondria. To explore the biochemical function of EG1, we tested its lipase activity [53] *in vitro* and found that both the full-length EG1 and truncated EG1 without predicted targeting peptides showed significant lipase activity (Fig 4E), indicating that EG1 encodes a functional lipase. Taken together, our results showed that EG1 functions as a predominately mitochondria-localized lipase.

### *EG1* functions in a high temperature-dependent manner in the floral robustness regulation

**Fig 5:**
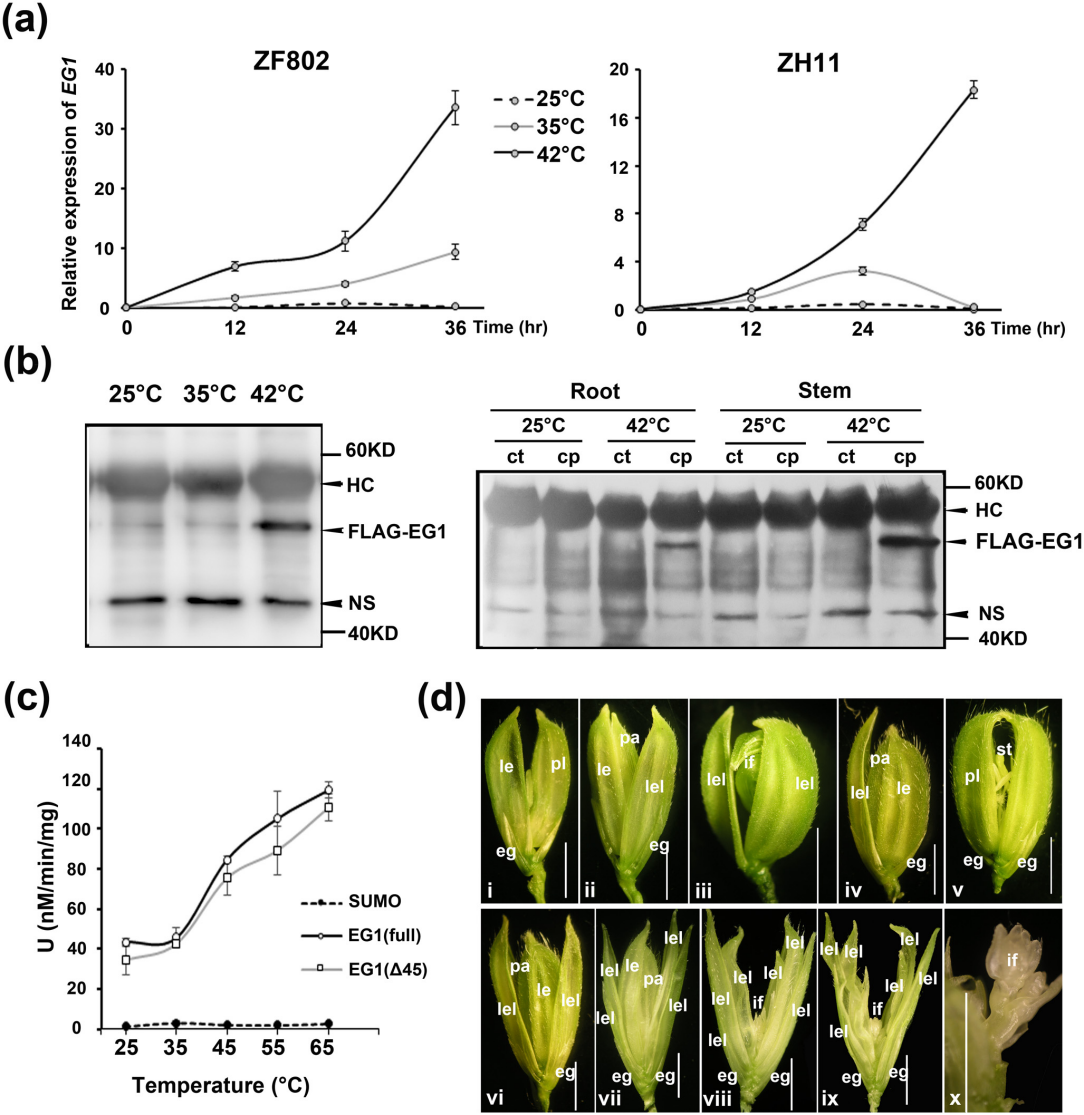
High temperature-dependent manner of *EG1* in floral robustness regulation. (a) RT-qPCR analysis of *EG1* expression induced by high temperatures in two wild-types. Values are means ± SE (n = 3), and significant difference was determined by ANOVA, *P < 0.05, **P < 0.01, and rice *α-TUBULIN* as the reference. (b) Western blot analysis of FLAG-EG1 protein accumulation under different temperatures and different tissues in the *EG1* complementation lines for 24 hr. Cp, Complementation lines; Ct, non-transgenic wild-type control. HC, Heavy chain of IgG; NS, Nonspecific band (as a loading control). (c) Temperature-dependent lipase activity of EG1. EG1 (Full) and EG1 (Δ45) respectively refer to full-length and no N-terminal (45 AA) protein of EG1 fused to SUMO peptide. Values are means ± SE for three independent experiments. (d) Floral phenotypes of *eg1* mutants in a condition of 40°C light 12 hr / 30°C dark 12 hr. Spikelets of *eg1-1* with pl, eg and rs phenotypes are shown on i, ii and iii, respectively. Spikelets of *eg1-2* with eg and pl phenotypes are shown on iv and v, and with multilayer lemma-like glumes (lel) and/or undetermined inflorescences primordia are on vi to x. x is the inside of ix. le, lemma; pl, palea-lemma mosaic organ; eg, empty glume; lel, lemma-like organ; pa, palea; st, stamen; if, inflorescence primordia. Bars = 2 mm.

The dependence of the *eg1* floral plasticity on environmental temperature raised a possibility that either *EG1* or its product or both are likewise regulated by temperature. To examine these possibilities, some heat/cold responsive cis-elements were discovered in the 2 kb genomic sequence upstream of the start codon of *EG1* (S3 Table), implying that its expression could be induced by extreme temperatures. To examine this possibility, one-week wild-type seedlings were treated under different temperatures and the *EG1* transcript was found to accumulate gradually, to an extremely high extent under heat shock (42°C) as well as usual high temperature 35°C for rice (Fig 5A), but to some extent suppressed under cold stress (4°C) (S7A Fig), indicating the high temperature-induced expression of *EG1*. A similar result was obtained by using young inflorescences in which *EG1* has a high expression (S7B Fig). To examine whether EG1 protein was also influenced by high temperatures, accumulation of FLAG-EG1 fusion protein in *eg1-2* complementation lines, with a temperature-insensitive promoter (S7C and S7E Fig), was detected under different temperatures and found it was significantly induced at extreme high temperature 42°C than 25°C and 35°C (Fig 5B and S7D Fig), indicating a stabilization of EG1 protein under heat stress. Furthermore, we detected that lipase activity of EG1 fusion proteins increase as temperature rising (Fig 5C), consistent with the assumption of EG1’s function required under high temperatures. Additionally, we also examined the effect of high temperatures on EG1 subcellular localization, and found no obvious translocation in protoplast system (S8A Fig), while failed to detect EG1 protein in the subcellular fractions of complementation lines except under heat stress for its minute amount (S8B Fig), suggesting that temperature does not significantly influence the subcellular localization of EG1. The increased the transcriptional level, protein stability and lipase activity of *EG1* under high temperatures, implying its more significant role under high temperatures. To verify this hypothesis, we observed the floral phenotypes of *eg1* mutants under extremely high temperatures and found much severer spikelets in *eg1* mutants especially in *eg1-2*, with multilayer lemma-like organs and undetermined inflorescence meristem, which have never been found in other temperature conditions (Fig 5D), showing a more significant function of *EG1* at higher temperatures in floral robustness. *eg1* was also found to grow faster than wild-type during primary growing days [54], and we detected this phenotype was much severer under extremely high temperature than others compared with wild-type, which was consistent with the floral phenotype (S9 Fig). Therefore, we concluded that *EG1* functions in a high temperature-dependent manner to regulate the floral robustness.

### *EG1* mediates the transcriptional responses of downstream genes and pathways to environmental fluctuation

**Fig 6:**
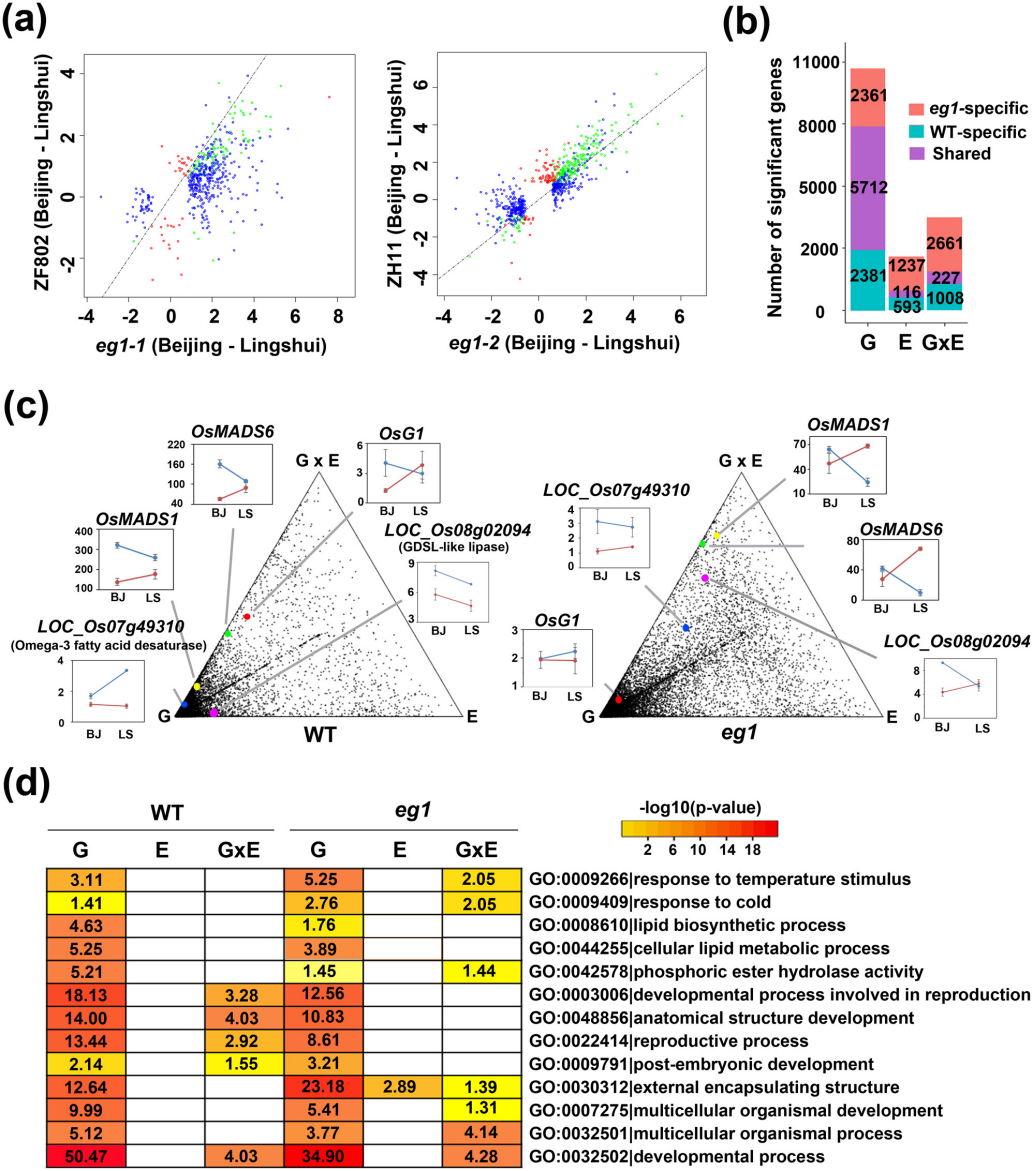
Genotype (G), environment (E) and genotype-environment interaction (GxE)-dependent gene expressional variations of *eg1*. (a) Scatterplots of comparisons of environmentally responsive genes between *eg1* alleles and wild-types. x and y axes are values of log2 [ratios of gene expression in Beijing to that in Lingshui] of two genotypes respectively. Points represent wild-type-specific (red), *eg1*-specific (blue) and shared (green) genes. Dotted lines indicate y = x lines. (b) Comparisons of genes significantly affected by G, E and GxE in wild-type and *eg1*. Effects were analyzed by two-way ANOVA. (c) Triangular scatterplots of distributions of genes significantly affected by G, E and GxE in wild-type and *eg1*. Each dot indicates a gene, and the three vertexes of triangle indicate three factors G, E, GxE respectively. The closer distance between a gene and a vertex means the stronger effect of the factor on the gene. Insets show expressions of some representative genes in ZF802/*eg1-1* (blue lines) and ZH11/*eg1-2* (red lines) of Beijing (BJ) and Lingshui (LS). Effects were analyzed by two-way ANOVA. (d) Comparisons of thirteen major pathways affected by G, E and GxE in wild-type and *eg1*. Numbers in boxes indicate -log10 (P-value) of the pathway enrichment tested by Fisher's exact test with Bonferroni correction and blank boxes no statistical significance.

Since the floral plasticity of *eg1* was influenced by both genotype and environment, to examine the molecular mechanism of genotype-environment interaction in floral plasticity, transcriptomes of inflorescences of two *eg1* alleles (*eg1-1* and *eg1-2*) and their wild-types (ZF802 and ZH11) in Beijing and Lingshui were analyzed. First, to evaluate the reliability of the transcriptomic data, we divided all transcripts into 33 modules by co-expression network analysis and analyzed their correlations with six variable phenotypes, and it turned out that the relationships among the variable phenotypes derived from these correlations were quite similar to their morphological correlations (S10 Fig), showing a good reliability of the transcriptomic data. Second, through overall comparisons of all transcriptomes, we discovered that the expression patterns of floral transcriptomes of *eg1* alleles between two environments were significantly different from their wild-types (S11A Fig), indicating a role of *EG1* in regulating expressions of environmentally responsive genes. The numbers of environmentally responsive genes in *eg1* mutants were much larger than that of wild-types, in contrast to the similar numbers between two wild-types or two *eg1* alleles (Fig 6A and S11B Fig), implying that *EG1* negatively regulates the responses of its downstream genes to environment. To verify this finding, we analyzed the effects of G, E, and GxE on transcriptomes of *eg1* and wild-type by two-way ANOVA and found that the number of genes significantly affected by E and GxE in *eg1* were significantly larger than that in wild-types (Fig 6B, S11C Fig and S4 Table), displaying a switch of many genes from G-affected to E/GxE-affected ones (Fig 6C), indicating that *EG1* represses its downstream genes not only to respond to, but also to interact with environment. Furthermore, the effects of the three factors on several important pathways varied significantly between *eg1* and wild-type, including the pathways related to temperature response, lipid metabolism and floral development (Fig 6D and S5 Table), indicating that *EG1* mediates a crosstalk of these pathways with environment. Since *EG1* was reported to regulate JA biosynthesis [54], in order to analyze its effect on the floral robustness control, we examined the expressional patterns of JA biosynthesis and signaling associated genes in our transcriptome data, and found that the transcriptional responsive patterns to environment or transcriptional level of several JA signaling genes (*JAZ7* and *JAZ8*) and JA biosynthesis genes (four methyltransferase genes) are varied in *eg1* mutants (S12 Fig), implying a possible role of JA in the *EG1*-associated floral robustness regulation but different from previously reported [54]. Taken together, these results revealed that *EG1* mediates the transcriptional responses of downstream genes and pathways to environmental fluctuation.

### *OsMADS1*, *OsMADS6* and *OsG1* act downstream of *EG1* to mediate floral robustness regulation

**Fig 7:**
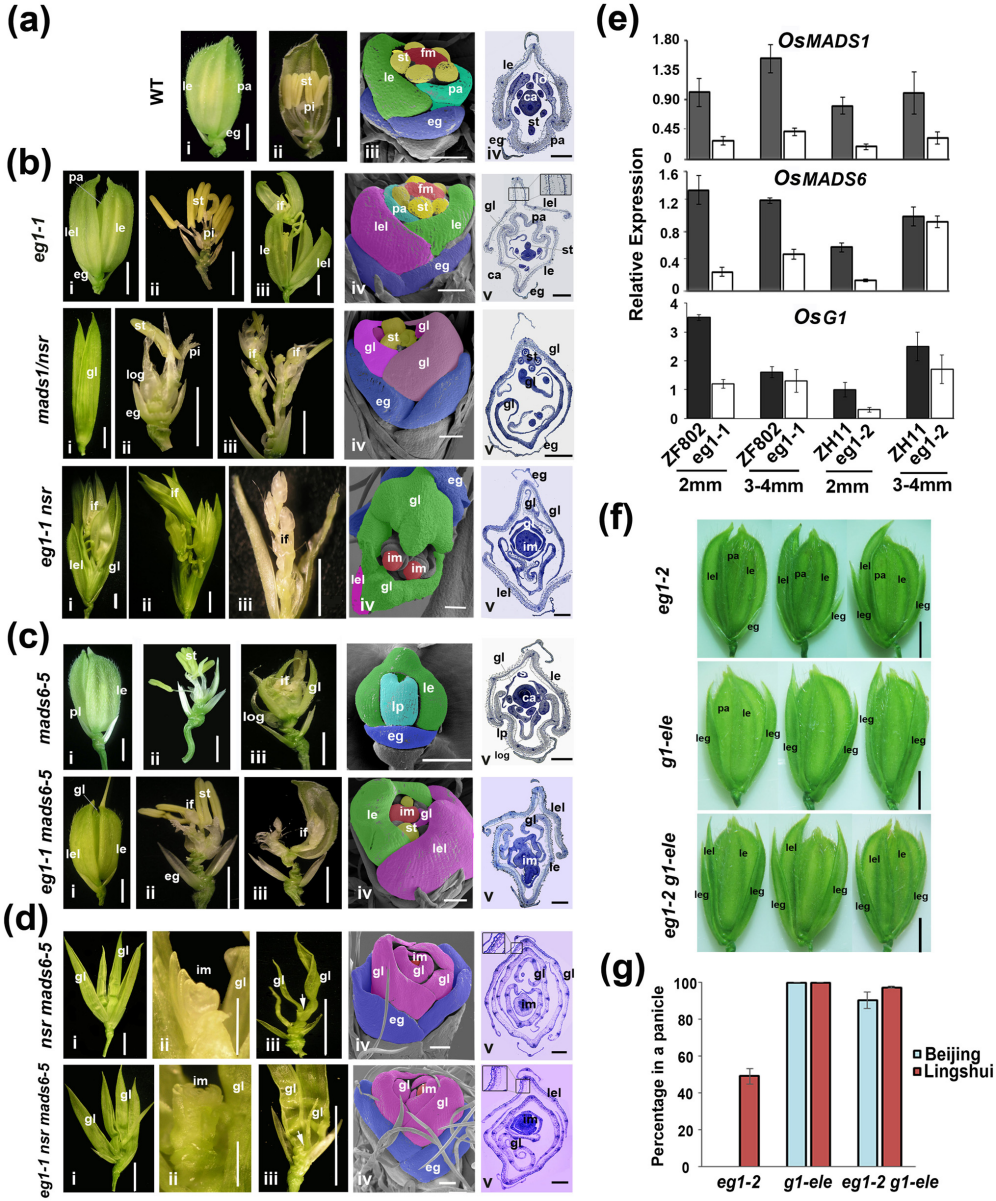
O*sMADS1*, *OsMADS6* and *OsG1* mediate the floral robustness regulation of *EG1*. (a) Spikelets of wild-type rice. i-iv show outside (i), inside (ii), SEM (iii) and paraffin transverse section (iv) of wild-type spikelets, respectively. (b) Genetic analysis of *eg1-1* and *mads1/nsr*. Photos show the outside (i), inside (ii-iii), SEM (iv) and paraffin transverse section (v) of *eg1-1*, *mads1/nsr* and *eg1-1 nsr* mutant spikelets, respectively. (c) Genetic analysis of *eg1-1* and *mads6-5*. Photos of *mads6-5* and *eg1-1 mads6-5* are shown as (b). (d) Genetic analysis of *eg1-1* and *nsr mads6-5*. Photos of *nsr mads6-5* and *eg1-1 nsr mads6-5* are shown as (b). Arrows on iii of *nsr mads6-5* and *eg1-1 nsr mads6-5* indicate the inactive growing points. (e) Relative expression levels of *OsMADS1*, *OsMADS6* and *OsG1* in wild-type and *eg1* inflorescences of different growth stages by RT-qPCR. Results are means ± SE. Rice *α-TUBULIN* was used as the reference. (f) Genetic analysis of *eg1* and *g1-ele*. Empty glume phenotypes of *eg1-2* in Lingshui, *g1-ele* and *eg1-2 g1-ele* in Beijing/Lingshui are shown respectively. (g) The phenotypic plasticity of le in *eg1-2*, *g1-ele* and *eg1-2 g1-ele*. Values are means ± SE, number of analyzed panicles ≥ 5. le, lemma; pa, palea; st, stamen; pi, pistil; eg, empty glume; ca, carpel; fm, floral meristem; im, inflorescence meristem; if, inflorescence primordia; lel, lemma-like organ; gl, glume-like organ; lp, lemma-palea mosaic organ; log, lodicule-glume mosaic organ. leg, long empty glume. Bars = 2 mm, 200 μm and 200 μm in spikelet structures, SEMs and paraffin transverse sections respectively, except for 0.5 mm in ii of (d).

The significant transcriptional effects on the floral development pathways based on the G, E and GxE analysis in *eg1* mutant suggested that floral identity genes are likely involved in *EG1*-dependent floral robustness regulation. To examine this, expressional variation of thirteen floral identity genes between two environments were further analyzed, and seven (*OsMADS1*, *OsMADS6*, *OsG1*, *OsMADS4*, *OsMADS7*, *OsMADS8*, *OsMADS58*) of them showed both varied environment-dependent expression patterns and repressed expression levels in *eg1* (S13 Fig), indicating their positive regulatory roles in the floral robustness. To further examine this possibility, genetic analysis between *EG1* and three of them (*OsMADS1*, *OsMADS6* and *OsG1*) were performed. *OsMADS1* and *OsMADS6* are two major genes regulating glume identity and floral determinacy in rice [61–69], and their expressions were significantly varied in *eg1* (Fig 7E and S13 Fig), indicating that *EG1* is required for their expressions. To examine their genetic relationships with *EG1*, a double mutant of *OsMADS1* mutant allele *nsr* [61] and *eg1-1* was generated and it exhibited longer and leafy lemmas/paleas similar to *nsr*, with all inner three whorls replaced by half-developed inflorescences or inflorescence primordia, which is severer than both single mutants (Fig 7B), indicating that *OsMADS1* functions downstream of *EG1* in lemma/palea identity and they may together regulate the determinacy of inner three whorls. In addition, the double mutant of *eg1-1* and *OsMADS6* mutant allele *osmads6-5* [66] showed abnormal paleas, with all transformed into one or two small lemma-like glumes and mostly with inflorescence primordia inside the spikelets (Fig 7C), indicating that *OsMADS6* functions downstream of *EG1* in specifying palea but may also regulate floral determinacy together with *EG1*. *osmads6-5* exhibited weaker floral disturbance in the F2 population when crossed with *eg1-1* (ZF802), with lemma-palea mosaic paleas and usually normal inner whorls (Fig 7C), showing its floral phenotype is also greatly influenced by genetic backgrounds. To further examine the relationships among *EG1*, *OsMADS1* and *OsMADS6* especially in floral determinacy, *nsr osmads6-5* and *eg1-1 nsr osmads6-5* were successively generated. The floral meristems of these two mutants similarly generated continuous glume primordia or became inactive before inner three whorls developed (Fig 7D), which were more dedifferentiated than the inflorescence primordia of *eg1-1 nsr* and *eg1-1 osmads6-5*, supporting the findings that both *OsMADS1* and *OsMADS6* act downstream of *EG1* and they together control the floral differentiation of inner three whorls. Compared with *eg1-1*, the rs plasticity of *eg1-1 nsr osmads6-5* totally disappeared when these two MADS genes were both deficient (Fig 7B and 7D), supporting the important roles of *OsMADS1* and *OsMADS6* in the rs plasticity regulation. Additionally, the spikelet phenotype of *eg1-1 nsr* [54] and *nsr osmads6-5* [68,69] were consistent to the previously reported, and *eg1* was linked with the lemma-like (lel) structure, and not affected by the deficiency of *OsMADS1* and *OsMADS6 (*Fig 7b–7d), implying that it is probably a special organ different from lemma and palea. Taken all these results together, we concluded that *MADS1* and *MADS6* together function downstream of *EG1* to control the determinacy of inner three whorls of rice floret as well as to mediate the rs plasticity of *eg1-1*.

Furthermore, the le phenotype (long empty glume) of *eg1-2* has the highest plasticity among all variable phenotypes (see Figs 1–3), and *OsG1* (*Long Sterile Lemma*) and *OsMADS19/34* are two crucial genes suppressing the elongation of empty glumes in rice [70–72]. The expression levels and patterns of *OsG1* but not *OsMADS19/34* appeared to be aberrant in *eg1-2* (Fig 7E and S13 Fig), implying that *OsG1* may contribute to the le phenotype of *eg1-2*. To confirm this, *g1*-*ele* allele of *OsG1* [72] was used to obtain a double mutant by crossing with *eg1-2*, and it turned out that almost all empty glumes of *eg1-2 g1-ele* were elongated to lemma-like organs similar to *g1*-*ele*, exhibiting much lower plasticity compared with *eg1-2* (Fig 7F and 7G), indicating that *OsG1* functions downstream of *EG1* in regulating empty glume development and contributes to the plastic le phenotype of *eg1-2*. Taken together, these results show that *OsMADS1*, *OsMADS6* and *OsG1* all act downstream of *EG1* to mediate the floral robustness regulation.

## Discussion

### *EG1* is a plasticity gene regulating the floral robustness against environmental fluctuation in rice

To our knowledge, no plasticity genes have been confirmed to function in floral robustness in flowering plants [47–52]. Given the sessile nature of plant species, uncovering this class of genes and dissecting their molecular mechanisms are crucial for understanding the biology of flower development and evolution. In this study, we have shown that *EG1* encodes a mitochondria-localized lipase functioning as a plasticity gene to regulate the rice floral robustness by a coordinated transcriptional regulation of temperature, lipid metabolism and flower development pathways. First, *eg1* alleles showed floral variations under both natural and artificial conditions and five *eg1* alleles produced increased floral plasticity. Second, RNA expression, protein stability and lipase activity of *EG1* can respond to environment enhancing its function significantly in severe conditions such as heat stress. Third, *EG1* appears to possess a “buffering” function of repressing environmental stimuli to interfere the target genes, and when environmental pressure becomes severer such as heat stress, the strengthened EG1 function induced by heat is enough to buffer the stronger and more deleterious effect of heat on the responsive transcriptional pathways. Last, *EG1* influences the expression of numerous floral identity genes, which are the direct contributors of plastic development of *eg1* spikelets. Thus, *EG1* is the first plasticity gene regulating plant floral robustness against environmental fluctuation. Our finding indicates that flowers retain a system containing *EG1* to sense and respond to environmental stimuli to maintain its stable development, and suggests a mechanism of transition from high plasticity to robustness in flower by recruiting plasticity-repressing genes, which ensures the coordinated development of organs with different plasticity in one organism.

### Mitochondria could serve as a hub for the lipid metabolism mediated-floral developmental robustness

**Fig 8:**
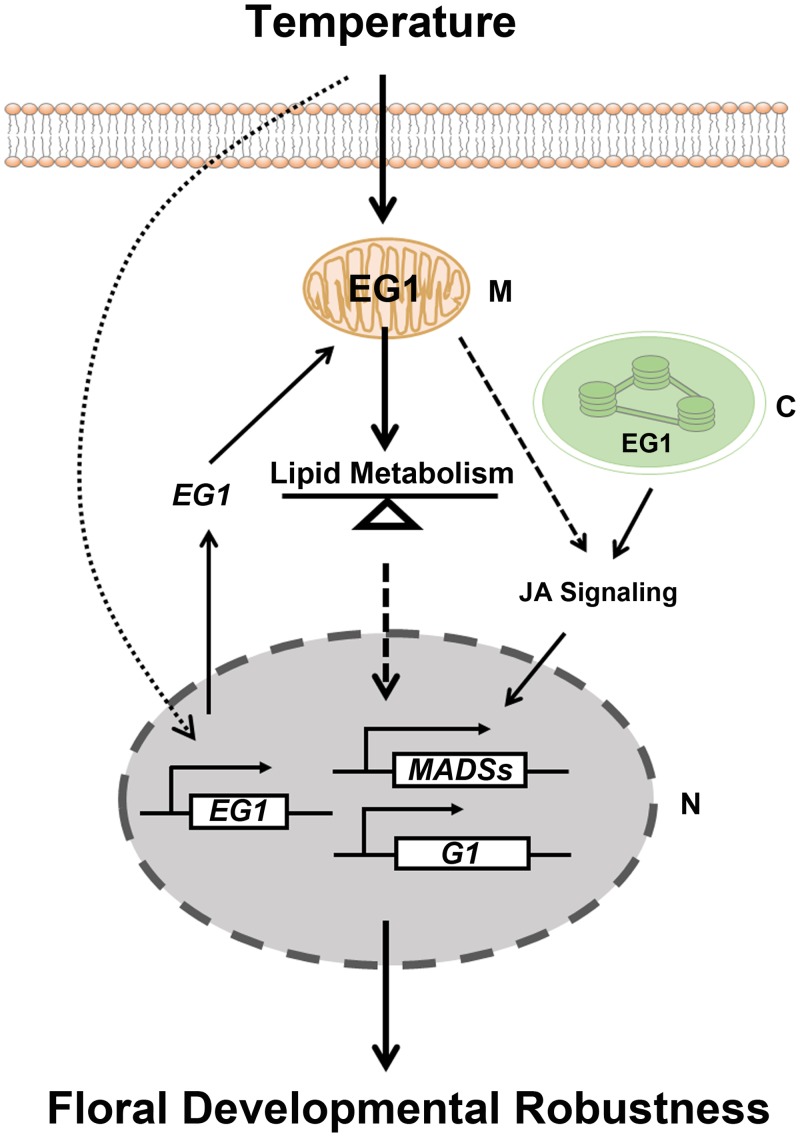
A model for *EG1* in regulating floral developmental robustness against temperature fluctuation in rice. *EG1* responses high temperature fluctuation at its transcriptional, posttranscriptional and lipase activity levels, and positively regulates the transcriptions of floral identity genes such as *OsMADS1*, *OsMADS6* and *OsG1*, which are mediated by a mitochondria-associated lipid metabolism, resulting in floral developmental robustness against temperature fluctuation. Additionally, chloroplasts-localized EG1 has also been shown to regulate the transcription of floral identity gene *OsMADS1* through JA signaling pathway [54].

Lipid metabolisms are known to be involved in adaptive plasticity of organisms [36,37,41,42], and mitochondria, as the energy factory of eukaryotes and one of subcellular compartmentations of lipid metabolism, have been reported to be crucial to the adaptive stability of plant vegetative traits [41,43,44]. As EG1 is a predominately mitochondria-localized functional lipase, our results suggest that the mitochondria-mediated lipid metabolism plays an important role in the regulation of floral robustness against temperature fluctuation. However, it remains unclear how this could be carried out. It is likely that mitochondria-related lipid homeostasis could serve as a “buffer” to relieve the effect of environmental stimuli and mediate the temperature-dependent floral plasticity regulation (Fig 8). Recently, *EG1* has been shown to influence JA synthesis, and JA signaling pathway regulates the transcription of floral identity gene *OsMADS1* [54], showing that JA potentially mediates the crosstalk of *EG1* and flower development related transcriptional factors. In fact, the most homologous gene of *EG1* in Arabidopsis *DGL* has been verified to function in JA biosynthesis [55,56,73], though its chloroplast location was questioned [55], suggesting that a non-chloroplast localized lipase is likely to function in JA production (Fig 8). On the other side, we may fail to detect EG1 protein due to its minute amounts in chloroplasts. Furthermore, according to the much severer phenotype of *eg1* and *eg2-1D* (a mutant allele of *EG2*/*OsJAZ1*) double mutant compared with two single [54], we noticed that JA signaling may be not the only pathway activated by *EG1* to mediate the signal transduction from outside to inside of nucleus in the *EG1*-associated floral regulation, other lipid-related pathways and regulatory mechanisms are also possible (Fig 8).

### Functional divergence of *EG1* and its homologs in angiosperms

In our study, *eg1* alleles showed a higher floral plasticity in *japonica* than that in *indica* varieties, while a severer floral disturbance in *indica*, revealing a functional divergence of *EG1* in rice subspecies. No differences in coding sequences but *cis*-elements of *EG1* in two subspecies were detected (S14 Fig), implying that this subspecific variation might be caused by an unknown differential responsive capability of promoters to environmental or endogenous stimuli. Besides, since genetic backgrounds are known to influence developmental outcomes via phenotypic modifiers [74,75], there also may be some *indica-*/*japonica*-specific modifiers to modulate *EG1* function due to their different genetic backgrounds, which can be either epistatic genes or specific lipid substrates of EG1 required for floral developmental robustness. Genetic dissection of these subspecific modifiers of plasticity will provide further insights into the molecular mechanism of floral development. So far, all reported *EG1* homologs in dicots have no apparent plasticity function in flowers [56–59], and *EG1* homologs in monocots can be mainly divided into two clades, one similar to that in dicots and the other unique to monocot species based on the phylogenetic tree and predicted subcellular localizations (S13 Fig), we thus speculate that *EG1* may have acquired a monocot-specific neofunctionalization in promoting floral robustness. Detailed genetic and biochemical analyses of these genes would provide additional clues to when and how the plasticity function and the subspecific divergence of *EG1* arose in monocot species.

In conclusion, our results revealed a novel function of *EG1* in floral developmental robustness against environmental fluctuation by mediating the mitochondrial lipid metabolism. Our finding also provide a genetic means to maintain the stable flower development under environmental stress ensuring grain yield stability in rice and potentially in other monocot species. Further studies should unlock the molecular crosstalk between mitochondria and nucleus in regulating floral developmental robustness.

## Materials and Methods

### Plant materials, growth conditions and spikelet phenotypic analysis

Five rice *eg1* recessive alleles were used in this study. *eg1-1* and *eg1-2* were from our previous work [53], in backgrounds of O. *sativa* L. spp. *indica* Zhefu 802 (ZF802) and O. *sativa* L. spp. *japonica* Zhonghua 11 (ZH11) respectively. *eg1-4* was generated from *indica* Dular, and *eg1-5* and *-6* from *japonica* Nipponbare by CRISPR/Cas9 technology [76,77]. Besides, ZF802>ZH11 and *eg1-1* (ZF802>ZH11) were isolated from an F2 population of ZF802 wild-type or *eg1-1* backcrossed with ZH11 three times, and ZH11>ZF802 and *eg1-2* (ZH11>ZF802) were isolated from an F2 population of ZH11 wild-type or *eg1-2* backcrossed with ZF802 once. Other rice mutants, *nsr* [61] and *g1-ele* [72] were kindly provided by Dr. Zhukuan Cheng, and *osmads6-5* also from our previous work [66].

Plants were grown in the natural conditions of Beijing (China) from Mar. to Oct. and Lingshui (Hainan province, China) from Dec. to Apr. (year 2014~2015). Weather data of 2014.7.28~2014.8.17 of Beijing and 2014.3.15~2014.4.4 of Lingshui, and 2014.2.1~2014.2.20 and 2014.4.1~2014.4.20 of Lingshui were shown respectively, which were ranged from ten days before spikelet meristem formation (~2 mm) to ten days after that. Artificial conditions in chambers were 35°C light 12 hr / 20°C dark 12 hr, 25°C light 12 hr / 20°C dark 12 hr and 40°C light 12 hr / 30°C dark 12 hr, respectively, with identical light intensity 50 μmol m^-2^ s^-1^ and relative air humidity 60%.

Spikelets of *eg1* were divided according to phenotypes of outer glumes (lemma, palea, empty glumes and extra glumes) following two rules: (1) the most significant mutant phenotype of *eg1* is in the outer glumes; (2) outer glumes have linkages with inner organs: normal palea always linked with normal inner organs, lemma-like palea with increased stamens and pistils, smaller palea with decreased stamens, multilayer glumes linked with rs. Main panicles/inflorescences of plants at booting stage were used for phenotypic analysis. Percentages of all variable phenotypes in one panicle were calculated for comparisons, and in some cases more than one phenotype appeared in a single spikelet. Data were statistically analyzed by using one/two-way ANOVA (Excel). Effects of genotype, environment and genotype-environment interaction on all variable phenotypes were calculated using phenotypic statistic data of *eg1* and wild-type grown in Beijing summer and Lingshui winter by two-way ANOVA. In CRISPR experiment, more than six independently homozygous lines were generated in each background, and their phenotypes were quite similar, thus only one/two lines were statistically analyzed and shown in our results.

### Protein subcellular localization analyses

Full-length *EG1* CDS was inserted into N- or C-terminal of *GFP* sequence of vector *pBI221-35SPro*:*GFP*, and full-length *EG1* CDS with 1.5 kb native promoter into *pCAMBIA1301*-*GFP*. *MTS-mOrange* and *Pro35S*:*COX11-GFP* plasmids were kindly provided by Dr. Yaoguang Liu [60], and the reported vector *pCAMBIA1301-35SPro*:*EG1-GFP* was provided by Dr. Dabing Zhang [54]. All plasmids of high quality were prepared for protoplast transfection. Rice protoplast preparation from 2-week-seedlings grown in light and polyethylene glycol (PEG)-mediated transfections were performed as described by Bart et al. [78]. Images were captured by a confocal microscope (FluoView 1000, Olympus).

Floral disorder of *eg1-2* was complemented by genetic transformation using vector *pTCK303-ProUBIQUTIN*:*FLAG-EG1*. One-week seedlings of *EG1* complementation lines were used for subcellular fractionation. Fractionations of mitochondria and chloroplasts were performed as described by Rodiger et al. [79]. After precipitating the organelle fractions, western blots were performed with α-FLAG (Sigma), mitochondria specific antibodies α-AOX1/2 and α-COXII (Agrisera), and chloroplasts specific antibodies α-RbcL and α-PsbA (Agrisera). Fractionation assays were performed with two independent complementation lines.

### Lipase activity analysis in vitro

Full-length *EG1* CDS and a truncated *EG1* CDS without predicted targeting sequence (135 bp) with engineered N-terminal SUMO tag were separately cloned into *pET-30a* (Novagen) to generate His6-SUMO-tagged fusion proteins. Mitochondrial targeting sequence was predicted with MitoProt II online [80]. *DGL* CDS without targeting sequence [81] was introduced into vector *PMAL-C2X* (NEB). All the fusion proteins were expressed in *E*. *coli* BL21 (DE3). The His_6_-SUMO-tagged fusion proteins were purified using Ni-NTA (Novagen) and eluted with buffer containing 25 mM Tris-HCl (pH 7.4), 150 mM NaCl and 250 mM imidazole. The MBP-tagged fusion proteins were purified using amylose resin (NEB) and eluted with buffer containing 50 mM Tris-HCl (pH 7.4) and 10 mM amylose. Imidazole in protein solutions was removed with desalting columns (Thermo Scientific) before lipase activity analysis.

p-nPB (p-nitrophenyl butyrate, Sigma) was used as the substrate of lipase analysis in vitro. Colorimetric assays for lipase activity of fusion proteins were performed as described by Seo et al. [82] with some modifications. A solution containing 1.11 mg/mL p-nPB (dissolved with isopropanol) and B solution containing 50 mM Tris-HCl (pH 7.4) and 0.1% Arabic gum were first prepared. Reactive solution was composed of 1 volume A solution and 9 volumes B solution with 2% Triton X-100. About 20 μl purified proteins (~5 μg) and 180 μl reactive solution were used for each reaction. After incubated under different temperatures for 30 min, p-nitrophenol formation from p-nitrophenyl butyrate was determined spectrophotometrically at 405 nm by an ELISA microplate reader. DGL and porcine pancreatic lipase (Sigma) were used for positive controls, and p-nitrophenol (Sigma) for the standard curve.

### Physiological experiments

*Cis*-elements in the genomic sequence upstream of the start codon of *EG1* was analyzed by PLACE online [83]. Wild-type ZF802, ZH11 and *EG1* complementation lines were grown at 25°C for one week after germination, and wild-type ZH11 seedlings were grown in a consistent condition till 2 mm inflorescence meristem developed before being treated under different temperatures for different time. The transcripts of *EG1* and *FLAG-EG1* were analyzed by RT-QPCR or RT-PCR. The root and shoot phenotypes of seedlings were statistically analyzed after growing under consistent temperatures for six days after germination. Other condition parameters of physiological experiments were daylight 12 hr, light intensity 50 μmol m^-2^ s^-1^ and relative air humidity 60%. We use 42°C as an extremely high temperature for short treat but 40°C for long treat by considering the tolerance of plants.

FLAG-EG1 protein in *EG1* complementation lines was enriched by immunoprecipitation for its small amount and detected by western blot with α-FLAG (Sigma).

### Environmental transcriptome analysis

Two mm inflorescence meristems of *eg1-1*, *eg1-2* and their wild-types ZF802 and ZH11 planted in Beijing summer and Lingshui winter were used for RNA sequencing, and two biological replicates were performed. Total RNAs were isolated with TRIzol kit (Invitrogen). Illumina sequencing libraries were prepared according to the manufacturer’s instructions (Illumina Part # 15026495Rev. D), and sequenced with Illumina system Hiseq2500. Analysis of RNA-seq data was conducted following the standard protocol as described by Trapnell et al. [84]. The raw reads of RNA-seq were mapped to MSU_IRGP_V7 (*japonica*) and Oryza_indicaASM465 v1.23 (*indica*) by Tophat [85]. Cuffdiff [85,86] was used to identify the differentially expressed genes between different genotypes or different environments. Co-expression network analysis was performed using R packages WGCNA. Enrichment pathways of genes significantly and specifically affected by G, E or GxE was analyzed and the -log10 (P-values) was tested by Fisher's exact test with Bonferroni correction as described by Lu et al. [87].

### Evolutionary analysis

Amino acids sequences of EG1 homologs in different plants were aligned with CLUSTAL W and maximum likelihood tree was constructed with MEGA6.0. Subcellular localizations of proteins were predicted with five sorts of software online. Among them, TargetP [88] (http://www.cbs.dtu.dk/services/TargetP/) has the best consistency compared with MitoProt II-v1.101 [80] (https://ihg.gsf.de/ihg/mitoprot.html), iPSORT [89] (http://ipsort.hgc.jp/), ProtComp 9.0 (http://linux1.softberry.com/berry.phtmtopic=protcompan&group=help&subgroup=proloc) and WoLF PSORT (http://www.genscript.com/wolf-psort.html) online. Predicted localizations with TargetP were shown in our results, a/b in which means the protein is more likely in “a” location than in “b” although both are possible.

## Supporting Information

S1 Fig Variable phenotypes of *eg1-1* and *-2*. Variable phenotypes of spikelets are defined as S1 Table. le, lemma; pa, palea; eg, empty glume; if, inflorescence primordia; sp, smaller pa; lel, lemma-like organ; pl, palea-lemma mosaic organ; leg, long empty glume in spikelet structures. Bars = 2 mm.
(TIF)

S2 Fig Mutations of five *eg1* alleles. The predicted full-length CDS (1308 bp) of *EG1* is shown. Horizontal red line indicates the sequence of lipase_3 domain, vertical black lines the mutation locations of alleles, and numbers in the bracket the numbers of bases from “A”.
(TIF)

S3 Fig Relative expressions of *EG1* in young inflorescences of several *indica* and *japonica* varieties. Values are means ± SE.
(TIF)

S4 Fig Local and seasonal variations of average daily temperatures during booting stage of rice. High/low temperature (T) per day during rice booting stage. Weather data of summer of Beijing and winter of Lingshui (a), or February and April of Lingshui (b) are shown. Values are means ± SD.
(TIF)

S5 Fig Co-transformation of *pCAMBIA1301-Pro35S-EG1-GFP* and *MTS-mOrange* in rice protoplasts. Green and red colors indicate the fluorescence of EG1-GFP and MTS-mOrange, respectively. Blue color indicates auto-fluorescence emitted by chloroplasts. DIC, pictures photographed by differential interference contrast microscope. Bar = 10 μm.
(TIF)

S6 Fig The normal spikelet and seed set rates in *EG1* complementation lines. Normal spikelet rate is the percentage of normal spikelets in a rice panicle, and seed set rate is the percentage of fully grown seeds in a panicle. Values are mean ± SE, number of analyzed panicles = 5.
(TIF)

S7 Fig Temperature-dependent gene expression of *EG1*. (a) RT-qPCR analysis of *EG1* expression in one-week seedlings of ZF802 and ZH11 wild-types treated under cold shock (4°C) for hours. Values are means ± SE (n = 3), and significant difference was determined by ANOVA, *P < 0.05, ** P < 0.01, and rice *α-TUBULIN* as the reference. (b) RT-qPCR analysis of *EG1* expression in the young inflorescence of ZH11 wild-type treated under extremely high temperature (42°C) for hours. (c) RT-PCR analysis of *FLAG-EG1* expression in one-week seedlings of *EG1* complementation lines treated under different temperatures. Two pairs of *FLAG-EG1* primers and three independent samples were used for analysis. Rice *α-TUBULIN* was for the reference. (d) Detection of FLAG-EG1 protein in *EG1* complementation lines at 35°C for 0, 3, or 5 days. HC, Heavy chain of IgG; NS, Nonspecific band (as a loading control). (e) Detection of GFP protein under three temperatures (25°C, 35°Cand 42°C) in the *ProUBIQUITIN-GFP* transgenic plants by western blot. RbcL was used as a loading reference. (f) Detection of the peptides derived from FLAG-EG1 by mass spectrometry. FLAG-EG1 was purified from one-week seedlings of *EG1* complementation lines and analyzed by MS. The peptides detected are shown in red letters.
(TIF)

S8 Fig Analysis of EG1 localization under different temperatures. (a) EG1-GFP localization under normal (28°C) or extremely high (40°C) temperatures in rice protoplasts. Mitochondria (Mit) and chloroplasts (Chl) are detected by Mito Tacker Red and auto-fluorescence. (b) Fractionation of mitochondria and chloroplasts in EG1 complementation line under different temperatures. Cp, Complementation lines; Ct, non-transgenic wild-type control. CB, Coomassie brilliant blue dyeing.
(TIF)

S9 Fig Seedling phenotype of *eg1* under different temperatures. (a) Phenotypes of six-day-old ZF802 and *eg1-1* seedlings. Bar = 1 cm. (b) Statistical analysis of root and shoot phenotypes of *eg1-1* and ZF802 seedlings. Values are means ± SE (n >20). Labelled values are ratios of average value of root length or height of *eg1-1* to that of ZF802 in the same condition. Significant difference was determined by ANOVA, *P < 0.05, ** P < 0.01.
(TIF)

S10 Fig Correlation analysis of the transcriptomic data and floral phenotypes of *eg1*. (a) The graph of correlations between gene modules and variable phenotypes. Each color represents a gene module (y axis), and six variable phenotypes are traits (x axis). The deeper colors in the middle squares show stronger correlations between modules and traits with positive correlations in red color and negative in green. Numbers in the boxes are correlation factors and P-values (inside brackets). (b) The network for correlations of gene modules and variable phenotypes. (c) Morphological correlation analysis of six floral variable phenotypes of *eg1* alleles.
(TIF)

S11 Fig Comparative and correlation analyses of expressional variations of environmentally responsive genes in *eg1* alleles. (a) Comparisons of environmentally responsive transcriptomes between two different genotypes. Each point represents a transcript. x and y axes are values of log2 [ratios of gene expression in Beijing to that in Lingshui] of two genotypes respectively. Dotted lines indicate y = x lines and solid lines are the best fit lines by linear regression. (b) Comparisons of environmentally responsive genes between two *eg1* alleles or two wild-types. Values of x and y axes are the same as (a). Points represent wild-type-specific (red), *eg1*-specific (blue) and shared (green) genes. Dotted lines indicate y = x lines. (c) Triangular scatterplot for distribution of total genes in wild-type and *eg1* affected by G, E and GxE. Each dot indicates a gene, and the three vertexes of triangle indicate three factors G, E, GxE respectively. The closer distance between a gene and a vertex means the stronger effect of the factor on the gene.
(TIF)

S12 Fig Expression patterns of 12 JA associated genes in the environmental transcriptomes. Genes with boldface letters indicate genes with significant variations of expression pattern between *eg1* and wild-type. Different colors indicate different samples of Beijing (BJ) and Lingshui (LS). Y axis indicates expression level of genes. Labelled values are log2 [ratios of gene mean expressions in BJ to that in LS] in the corresponding genotypes. “*” labels |log2|>1.
(TIF)

S13 Fig Expression patterns of a total of 13 floral identity genes in the environmental transcriptomes. Column diagrams are described as in S12 Fig.
(TIF)

S14 Fig Maximum likelihood tree and predicted subcellular locations of EG1 and its homologs in different species. Proteins from dicots: *Arabidopsis thaliana* (AtDGL/At1g05800, At2g31690, AtDLAH/At1g30370, AtDSEL/At4g18550, AtDAD1/At2g44810), *Glycine max* (Glyma.08G362700, Glyma.18G299300), *Ricinus communis* (28424.t000001(Rc)) and *Medicago truncatula* (Medtr7g006400); from monocots: *Oryza sativa* (LOC_Os11g19340, LOC_Os11g19290, OsEG1, LOC_Os01g67450, LOC_Os02g42170), *Brachypodium distachyon* (Bradi4g20220, Bradi4g20230, Bradi2g57760), *Zea mays* (GRMZM2G117627_T0, GRMZM5G812425_T01, GRMZM2G406951_T01), *Sorghum bicolor* (Sobic.005G111400, Sobic.007G187000, Sobic.003G390900, Sobic.003G391000); and moss *Physcomitrella patens* (Phpat.022G001500). Gene IDs come from JGI website. chlo, chloroplast; mito, mitochondria; other, without a chlo/mito targeting peptide.
(TIF)

S1 Table Description of variable phenotypes of *eg1* alleles. (XLSX)

S2 Table Differences of PLACE elements in the 4 kb putative promoters of *EG1* in different genetic backgrounds. (XLSX)

S3 Table Temperature-related cis-regulatory elements in the 2kb promoter of *EG1*. (XLSX)

S4 Table Genes significantly affected by G, E and GxE in wild-type and *eg1*. (XLSX)

S5 Table Genes of thirteen major pathways significantly affected by G, E and GxE in wild-type and *eg1*. (XLSX)

S6 Table Predicted subcellular localizations of EG1 and its homologs with five online softwares. (XLSX)

S7 Table Primers for genotyping the mutants. (XLSX)

S8 Table Primers for vector constructions. (XLSX)

S9 Table Primers for RT-QPCR or RT-PCR. (XLSX)
