# Supplementary figures and images for: A High Temperature-Dependent Mitochondrial Lipase EXTRA GLUME1 Promotes Floral Phenotypic Robustness against Temperature Fluctuation in Rice (Oryza sativa L.)
Source: PLoS Genet. 2016 Jul 1;12(7):e1006152. doi: 10.1371/journal.pgen.1006152 (PMC4930220; doi:10.1371/journal.pgen.1006152)

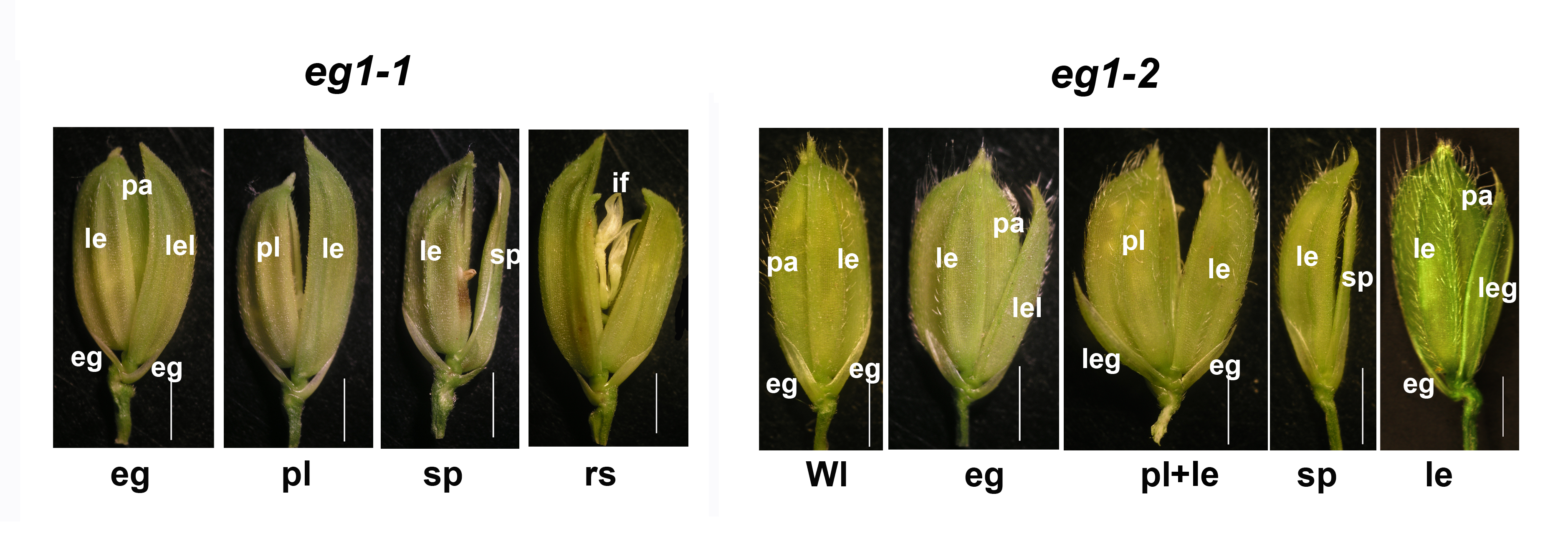

Supplement: S1 Fig — Variable phenotypes of spikelets are defined as S1 Table. le, lemma; pa, palea; eg, empty glume; if, inflorescence primordia; sp, smaller pa; lel, lemma-like organ; pl, palea-lemma mosaic organ; leg, long empty glume in spikelet structures. Bars = 2 mm. (TIF) [file pgen.1006152.s001.tif]

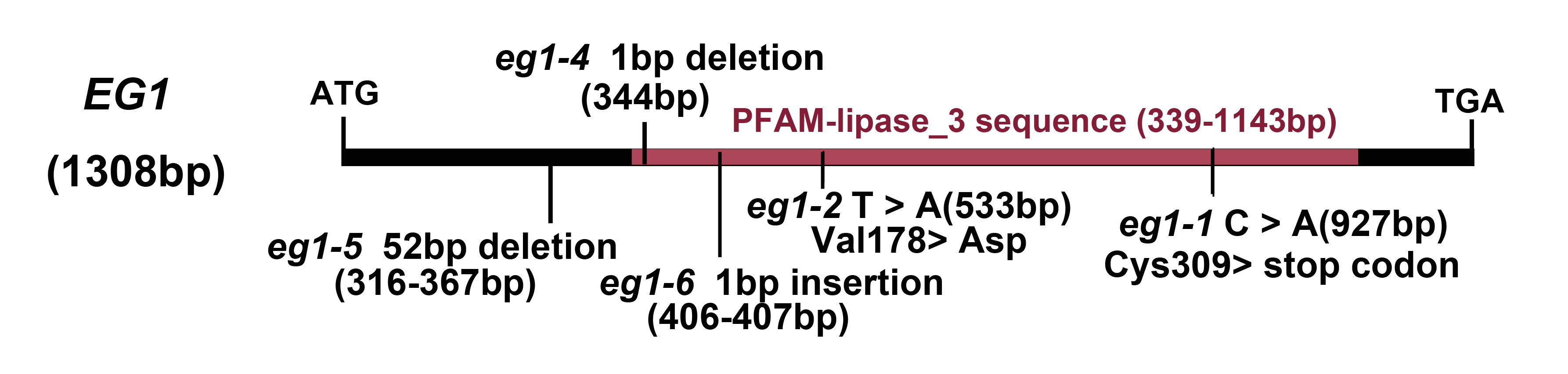

Supplement: S2 Fig — The predicted full-length CDS (1308 bp) of EG1 is shown. Horizontal red line indicates the sequence of lipase_3 domain, vertical black lines the mutation locations of alleles, and numbers in the bracket the numbers of bases from “A”. (TIF) [file pgen.1006152.s002.tif]

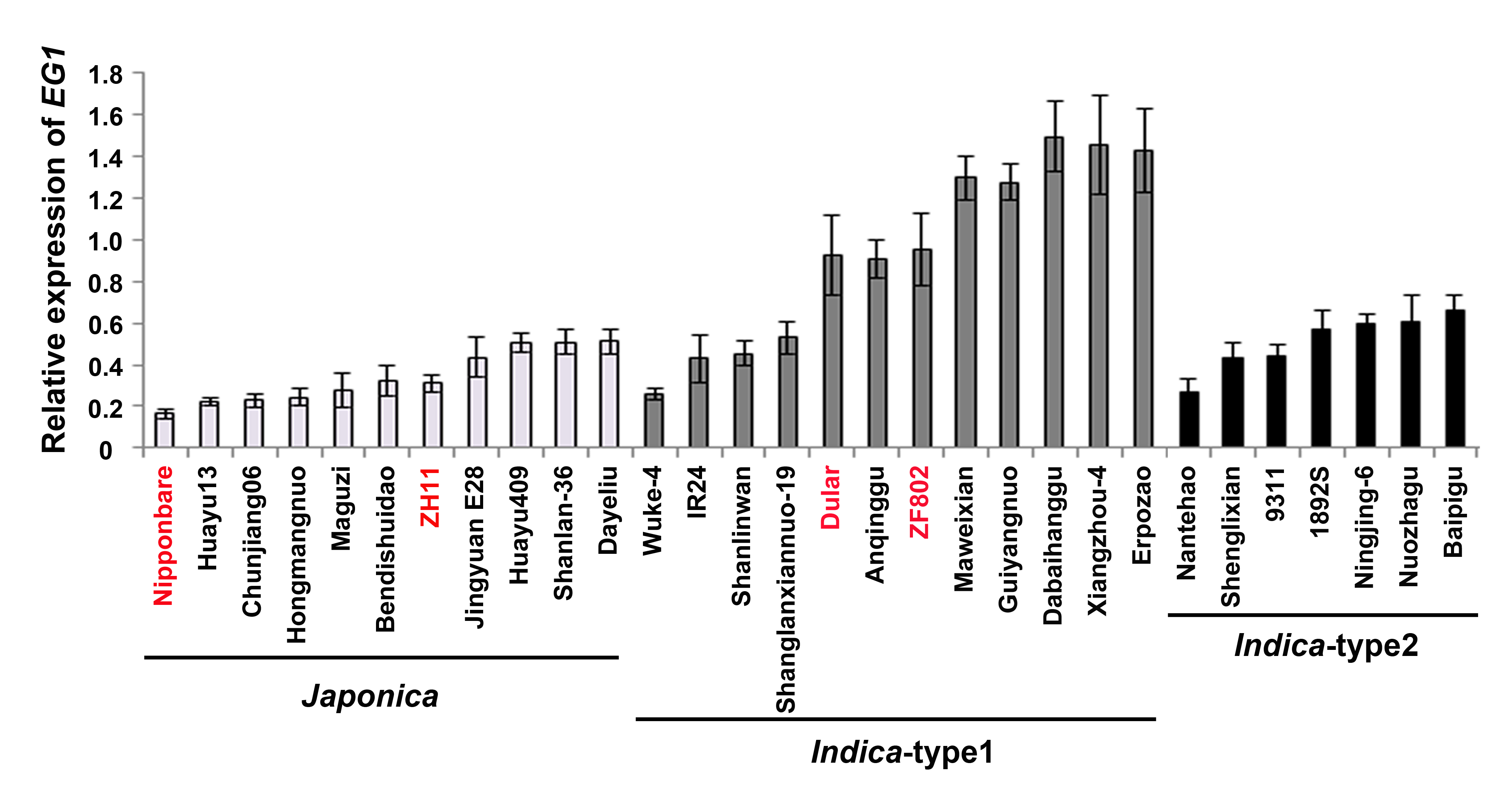

Supplement: S3 Fig — Values are means ± SE. (TIF) [file pgen.1006152.s003.tif]

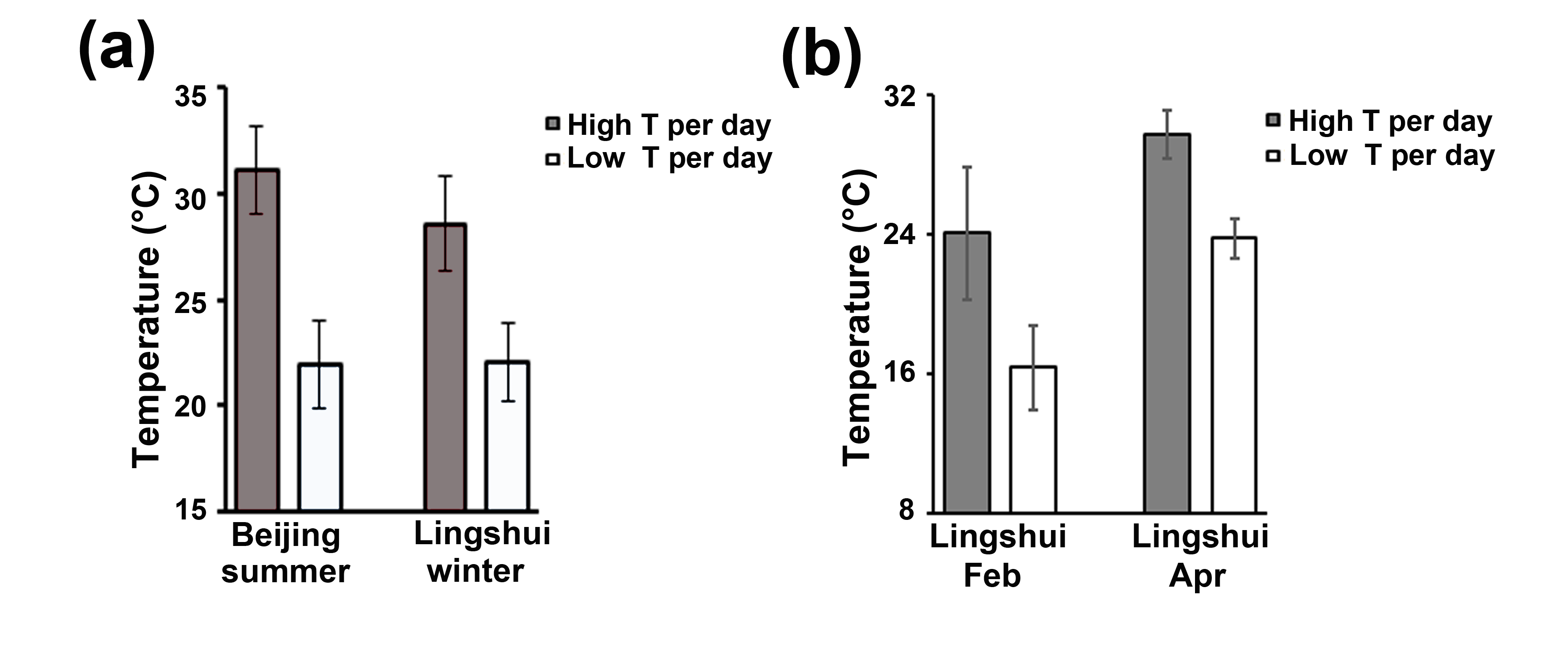

Supplement: S4 Fig — High/low temperature (T) per day during rice booting stage. Weather data of summer of Beijing and winter of Lingshui (a), or February and April of Lingshui (b) are shown. Values are means ± SD. (TIF) [file pgen.1006152.s004.tif]

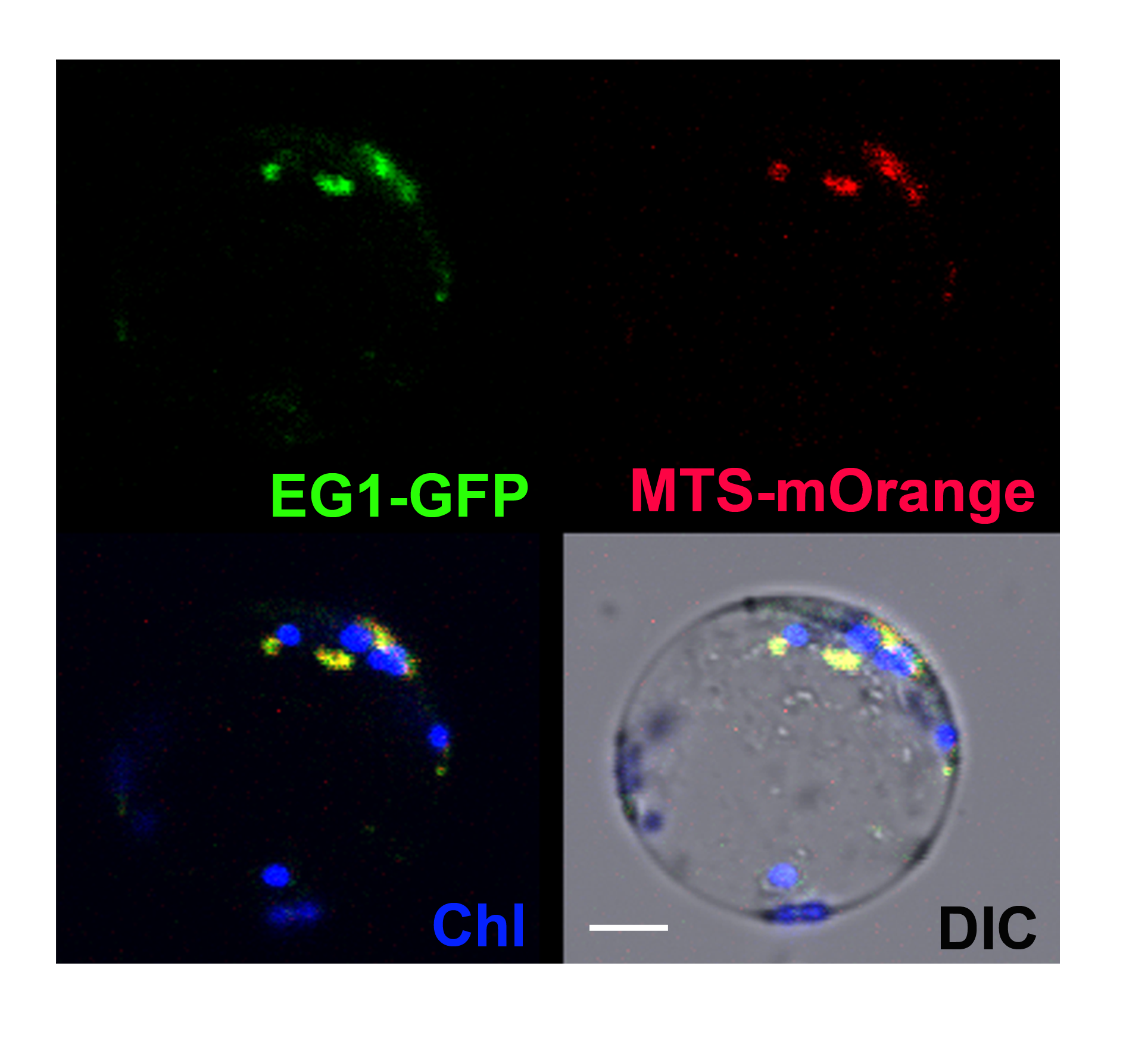

Supplement: S5 Fig — Green and red colors indicate the fluorescence of EG1-GFP and MTS-mOrange, respectively. Blue color indicates auto-fluorescence emitted by chloroplasts. DIC, pictures photographed by differential interference contrast microscope. Bar = 10 μm. (TIF) [file pgen.1006152.s005.tif]

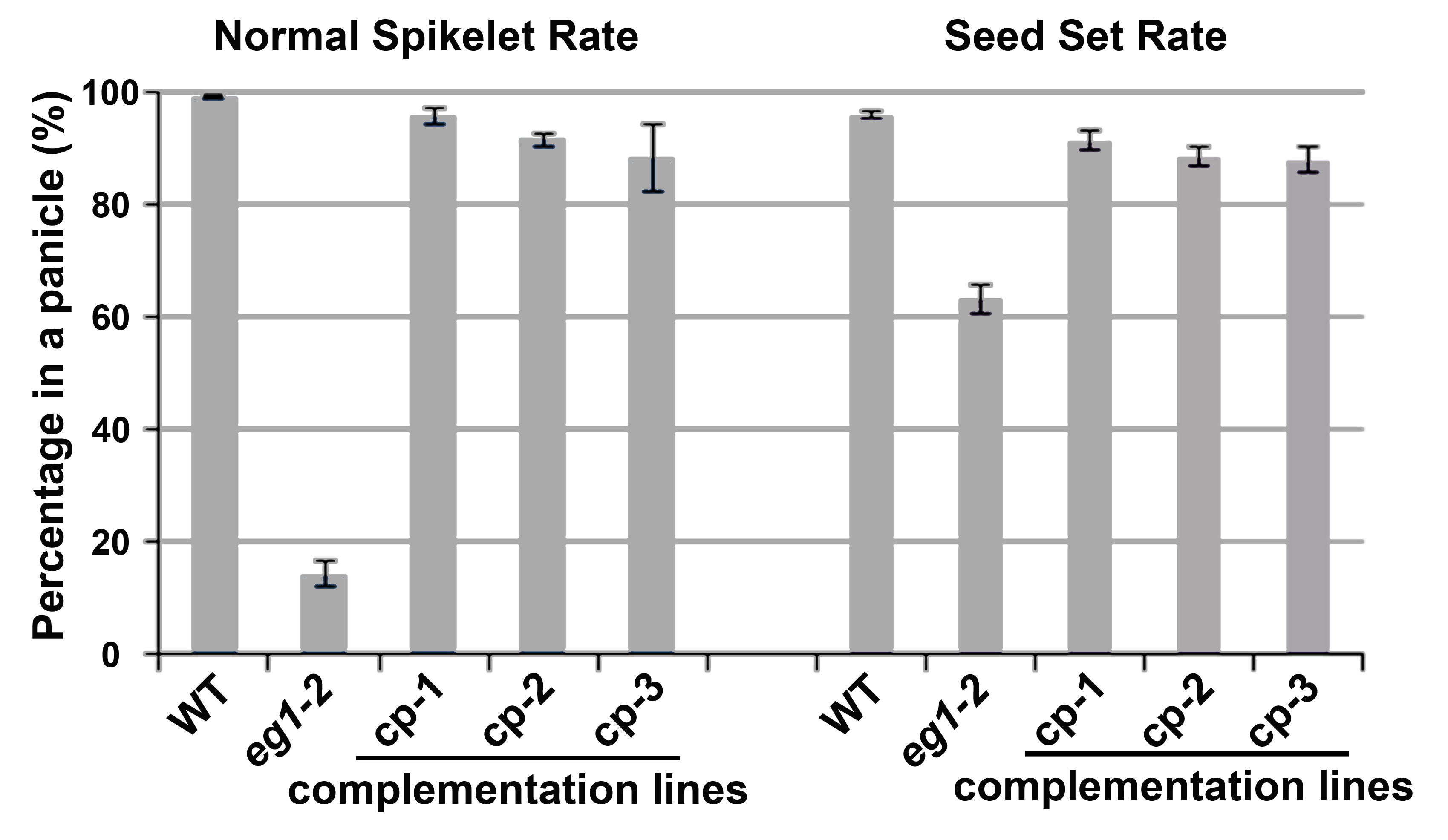

Supplement: S6 Fig — Normal spikelet rate is the percentage of normal spikelets in a rice panicle, and seed set rate is the percentage of fully grown seeds in a panicle. Values are mean ± SE, number of analyzed panicles = 5. (TIF) [file pgen.1006152.s006.tif]

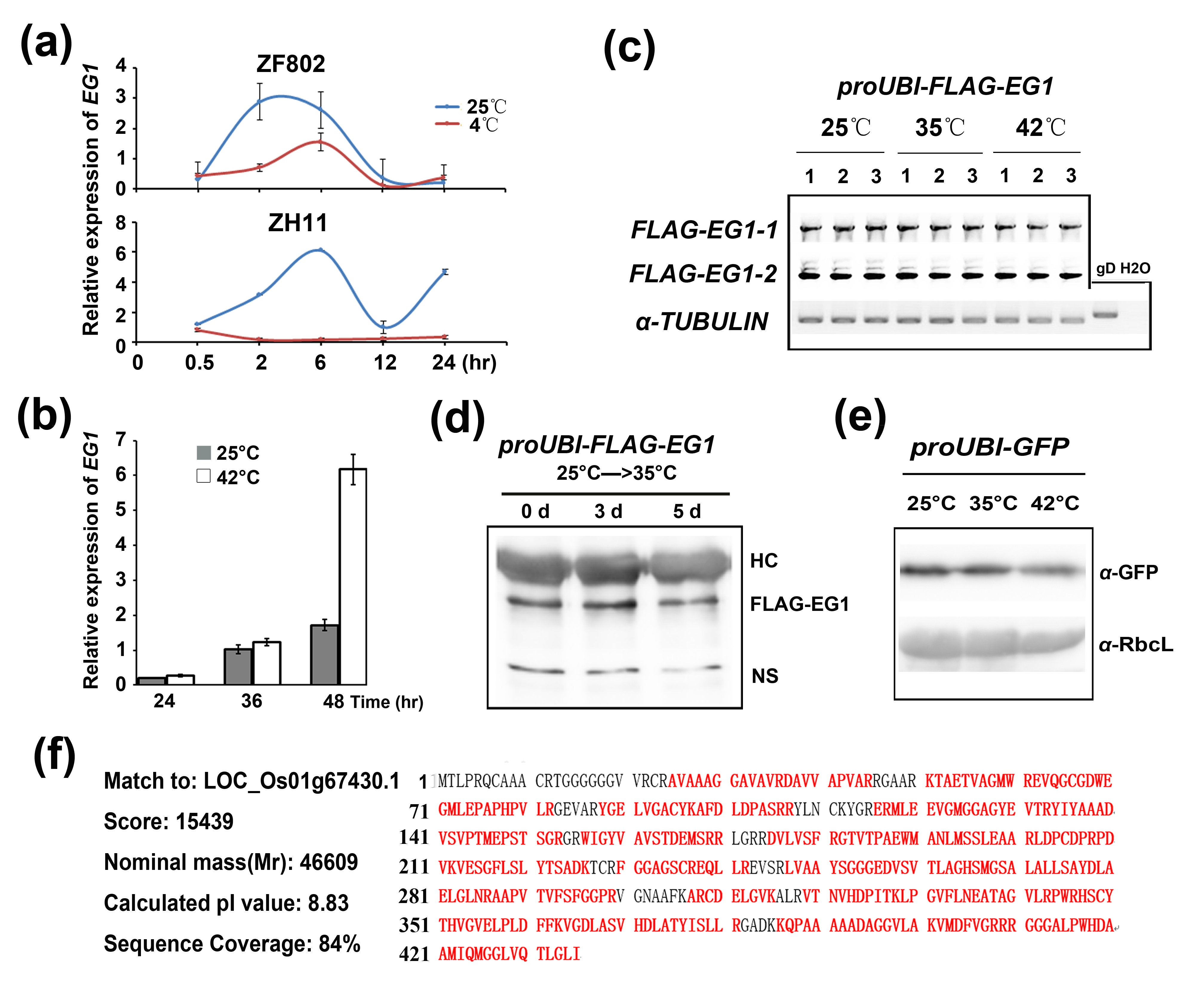

Supplement: S7 Fig — (a) RT-qPCR analysis of EG1 expression in one-week seedlings of ZF802 and ZH11 wild-types treated under cold shock (4°C) for hours. Values are means ± SE (n = 3), and significant difference was determined by ANOVA, *P < 0.05, ** P < 0.01, and rice α-TUBULIN as the reference. (b) RT-qPCR analysis of EG1 expression in the young inflorescence of ZH11 wild-type treated under extremely high temperature (42°C) for hours. (c) RT-PCR analysis of FLAG-EG1 expression in one-week seedlings of EG1 complementation lines treated under different temperatures. Two pairs of FLAG-EG1 primers and three independent samples were used for analysis. Rice α-TUBULIN was for the reference. (d) Detection of FLAG-EG1 protein in EG1 complementation lines at 35°C for 0, 3, or 5 days. HC, Heavy chain of IgG; NS, Nonspecific band (as a loading control). (e) Detection of GFP protein under three temperatures (25°C, 35°Cand 42°C) in the ProUBIQUITIN-GFP transgenic plants by western blot. RbcL was used as a loading reference. (f) Detection of the peptides derived from FLAG-EG1 by mass spectrometry. FLAG-EG1 was purified from one-week seedlings of EG1 complementation lines and analyzed by MS. The peptides detected are shown in red letters. (TIF) [file pgen.1006152.s007.tif]

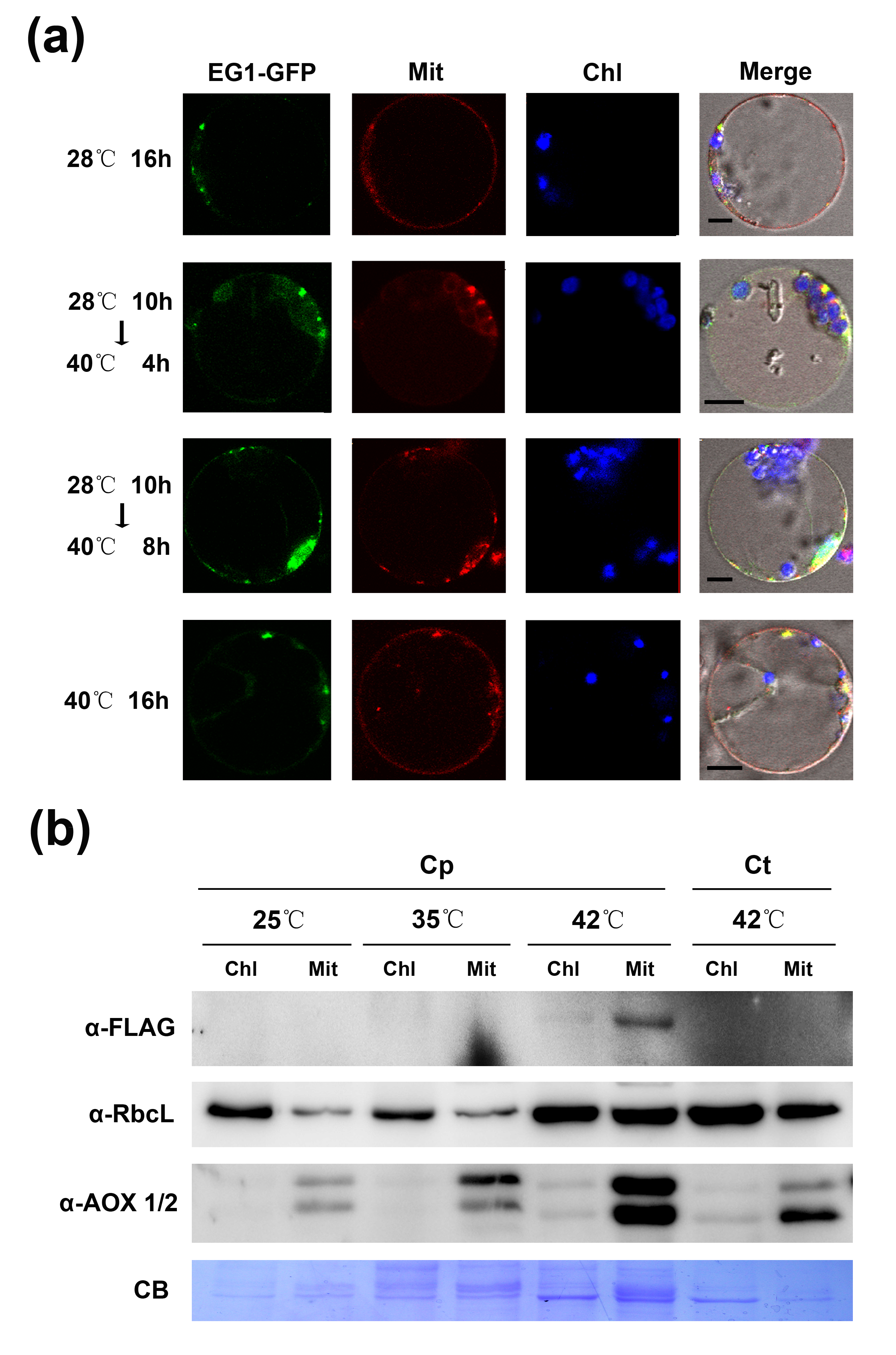

Supplement: S8 Fig — (a) EG1-GFP localization under normal (28°C) or extremely high (40°C) temperatures in rice protoplasts. Mitochondria (Mit) and chloroplasts (Chl) are detected by Mito Tacker Red and auto-fluorescence. (b) Fractionation of mitochondria and chloroplasts in EG1 complementation line under different temperatures. Cp, Complementation lines; Ct, non-transgenic wild-type control. CB, Coomassie brilliant blue dyeing. (TIF) [file pgen.1006152.s008.tif]

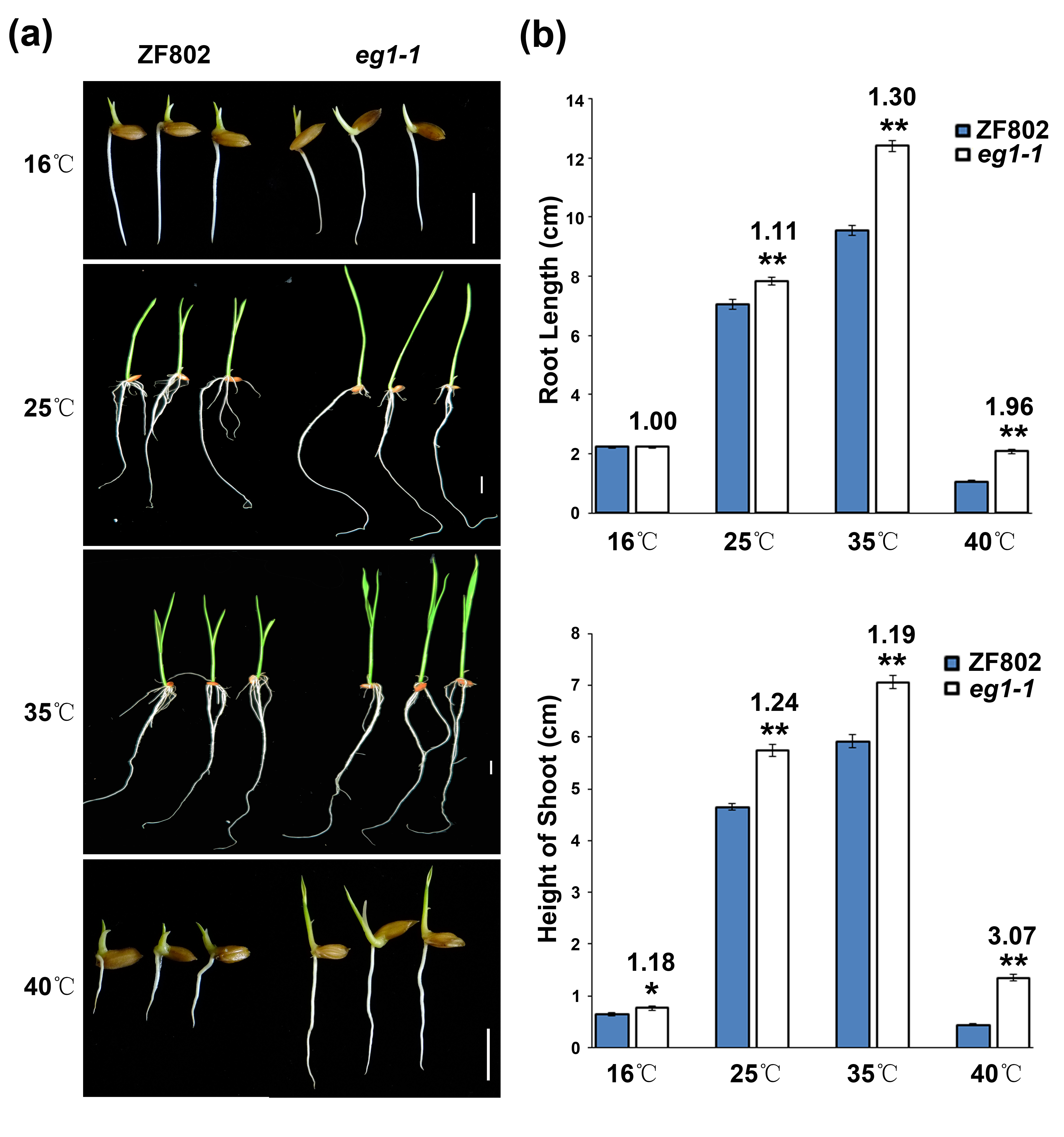

Supplement: S9 Fig — (a) Phenotypes of six-day-old ZF802 and eg1-1 seedlings. Bar = 1 cm. (b) Statistical analysis of root and shoot phenotypes of eg1-1 and ZF802 seedlings. Values are means ± SE (n >20). Labelled values are ratios of average value of root length or height of eg1-1 to that of ZF802 in the same condition. Significant difference was determined by ANOVA, *P < 0.05, ** P < 0.01. (TIF) [file pgen.1006152.s009.tif]

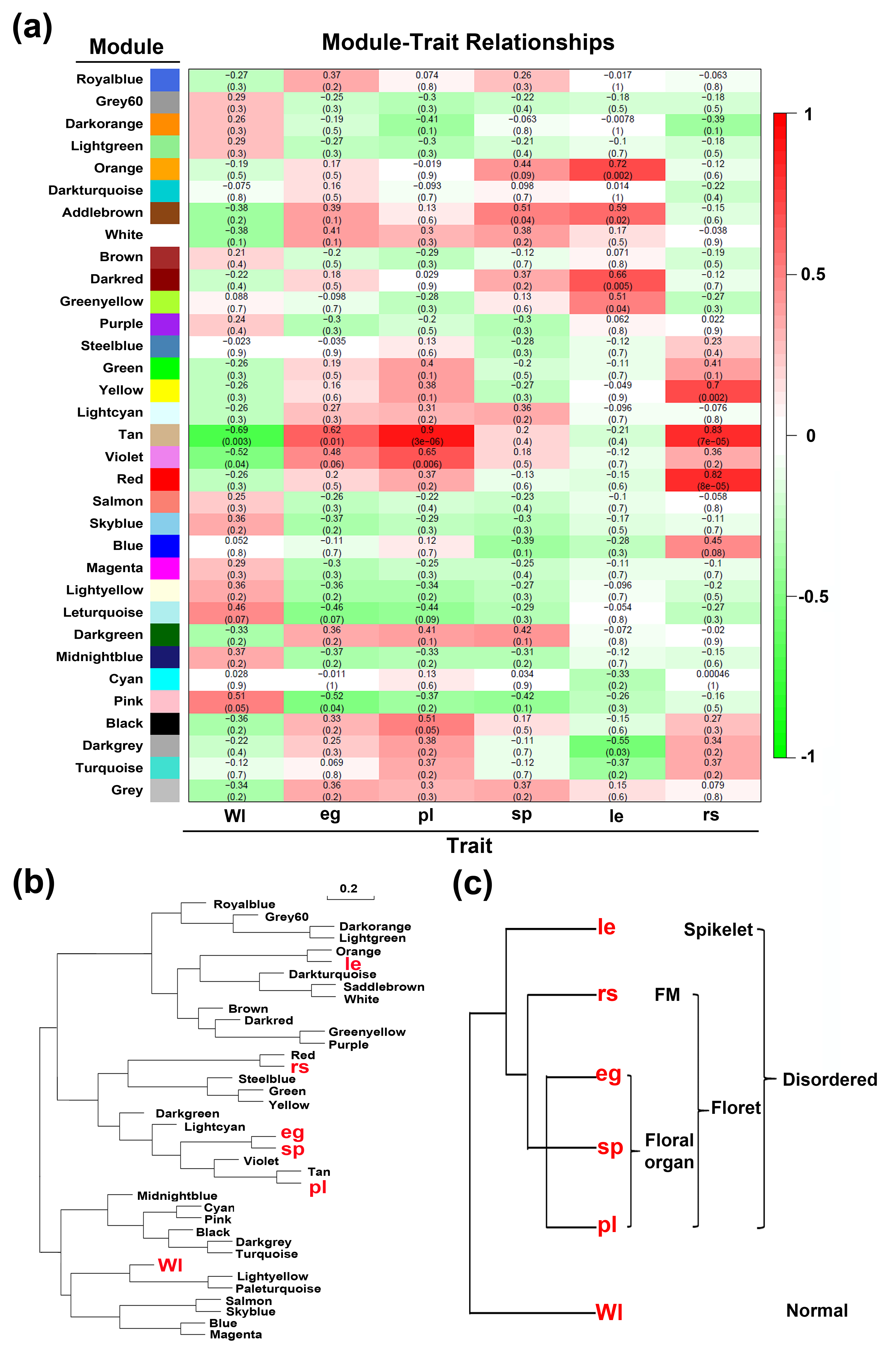

Supplement: S10 Fig — (a) The graph of correlations between gene modules and variable phenotypes. Each color represents a gene module (y axis), and six variable phenotypes are traits (x axis). The deeper colors in the middle squares show stronger correlations between modules and traits with positive correlations in red color and negative in green. Numbers in the boxes are correlation factors and P-values (inside brackets). (b) The network for correlations of gene modules and variable phenotypes. (c) Morphological correlation analysis of six floral variable phenotypes of eg1 alleles. (TIF) [file pgen.1006152.s010.tif]

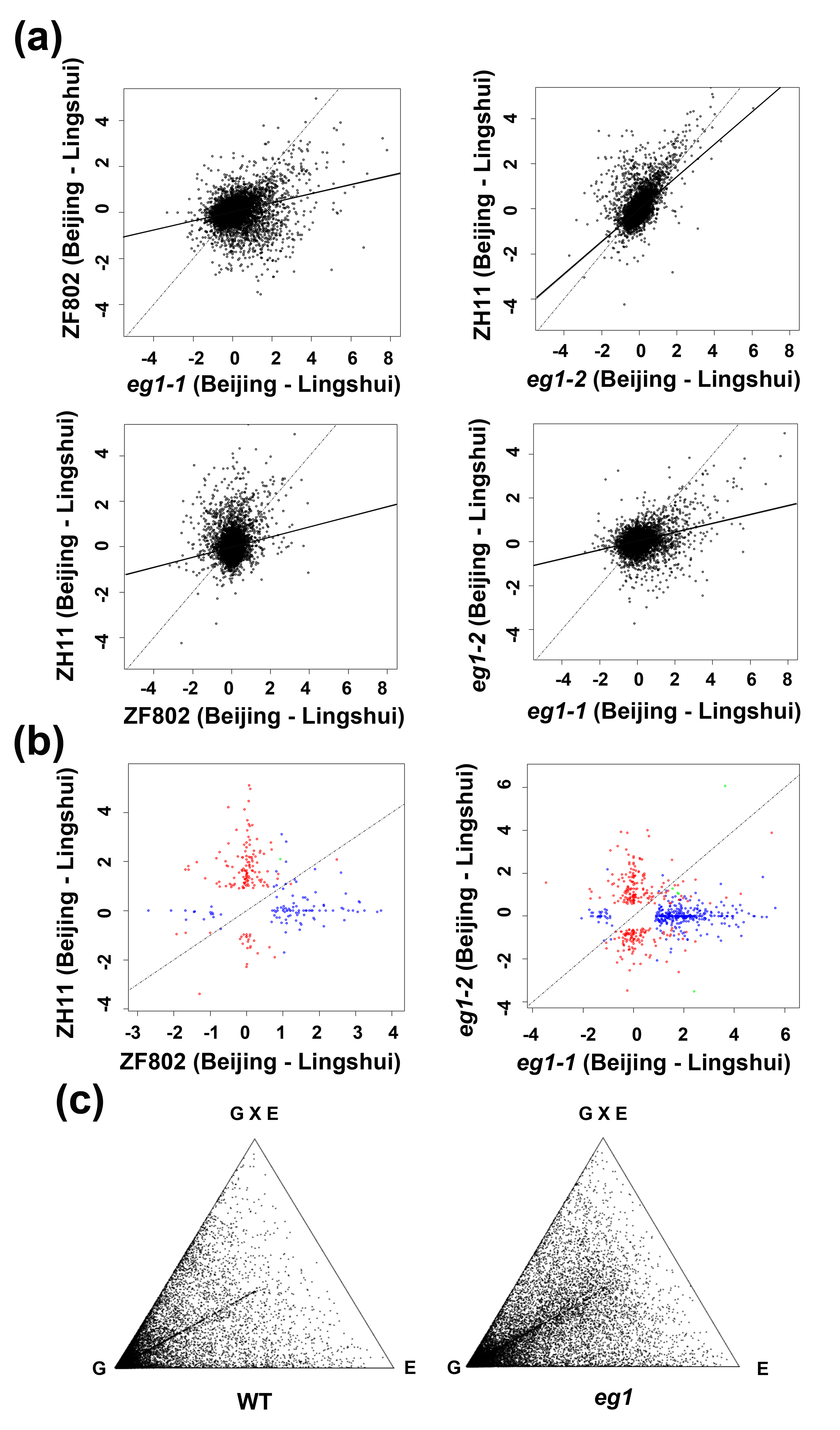

Supplement: S11 Fig — (a) Comparisons of environmentally responsive transcriptomes between two different genotypes. Each point represents a transcript. x and y axes are values of log2 [ratios of gene expression in Beijing to that in Lingshui] of two genotypes respectively. Dotted lines indicate y = x lines and solid lines are the best fit lines by linear regression. (b) Comparisons of environmentally responsive genes between two eg1 alleles or two wild-types. Values of x and y axes are the same as (a). Points represent wild-type-specific (red), eg1-specific (blue) and shared (green) genes. Dotted lines indicate y = x lines. (c) Triangular scatterplot for distribution of total genes in wild-type and eg1 affected by G, E and GxE. Each dot indicates a gene, and the three vertexes of triangle indicate three factors G, E, GxE respectively. The closer distance between a gene and a vertex means the stronger effect of the factor on the gene. (TIF) [file pgen.1006152.s011.tif]

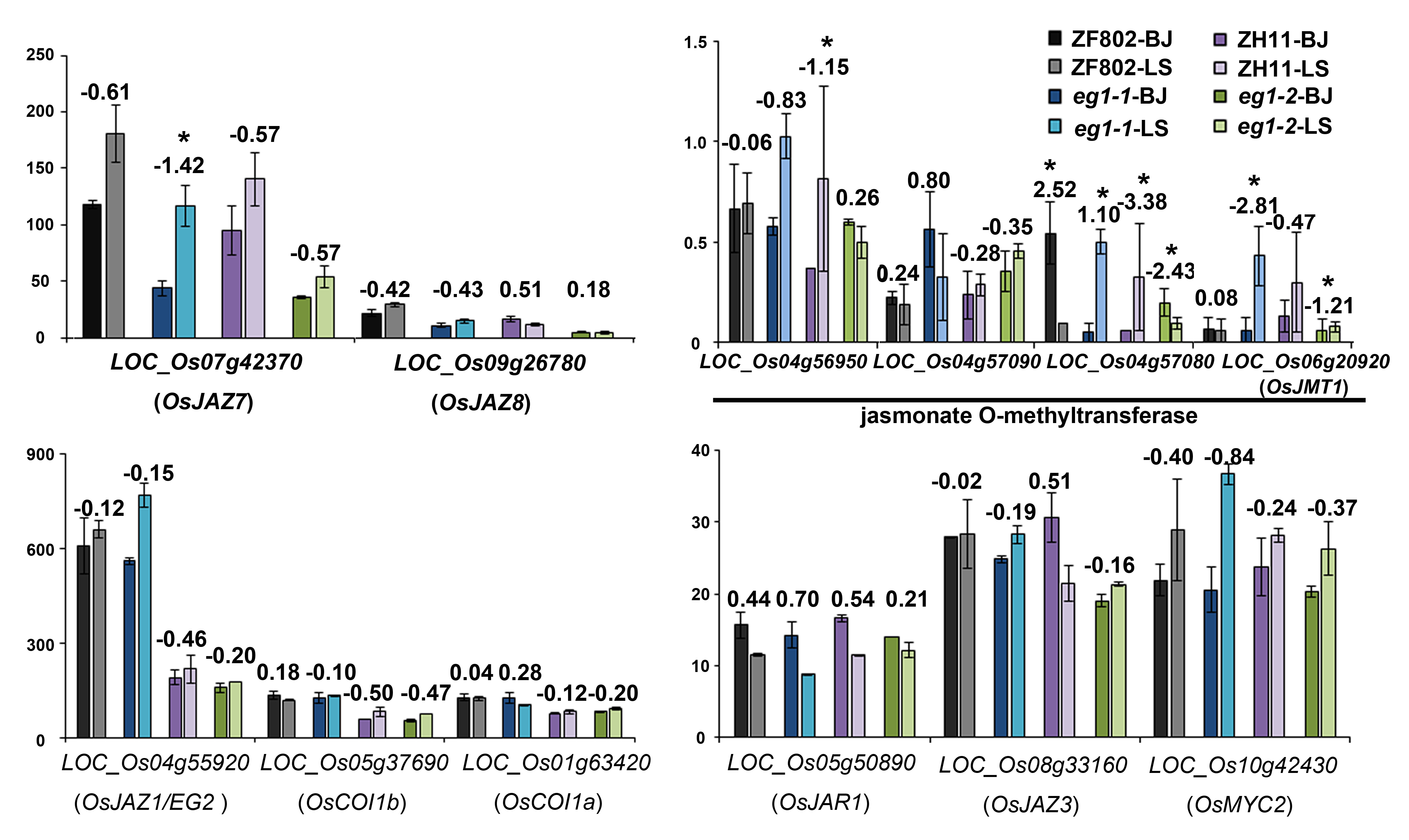

Supplement: S12 Fig — Genes with boldface letters indicate genes with significant variations of expression pattern between eg1 and wild-type. Different colors indicate different samples of Beijing (BJ) and Lingshui (LS). Y axis indicates expression level of genes. Labelled values are log2 [ratios of gene mean expressions in BJ to that in LS] in the corresponding genotypes. “*” labels |log2|>1. (TIF) [file pgen.1006152.s012.tif]

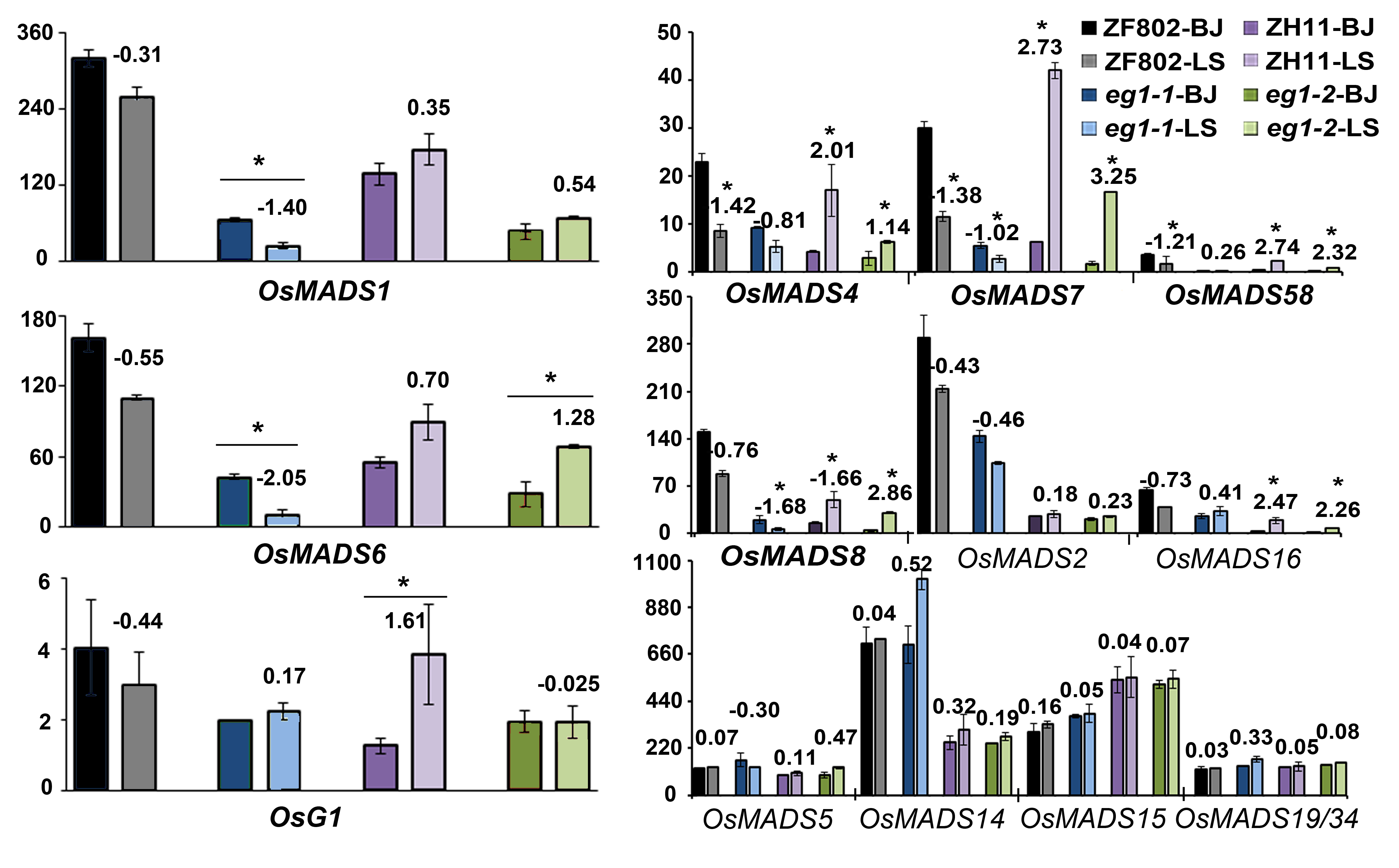

Supplement: S13 Fig — Column diagrams are described as in S12 Fig. (TIF) [file pgen.1006152.s013.tif]

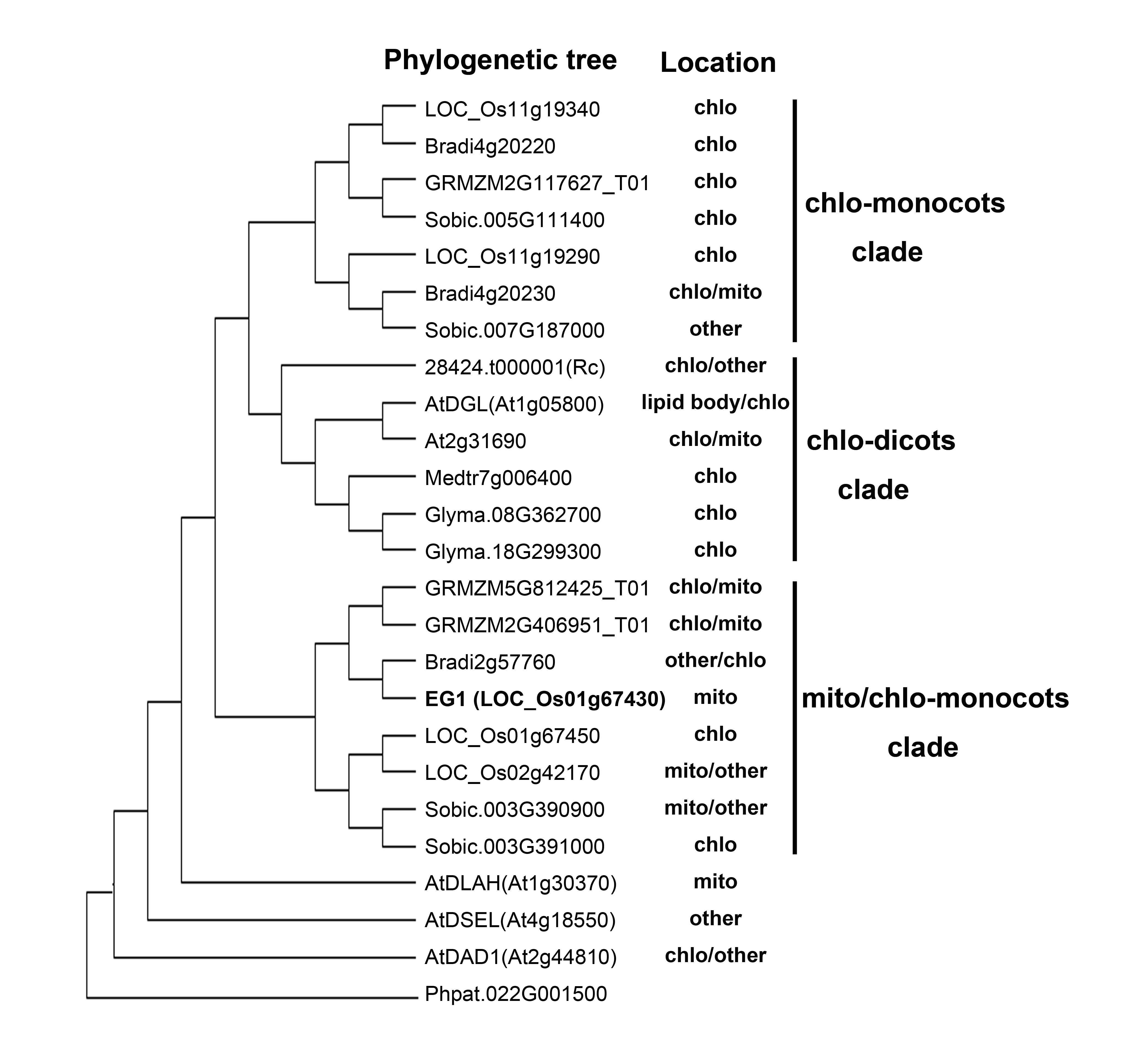

Supplement: S14 Fig — Proteins from dicots: Arabidopsis thaliana (AtDGL/At1g05800, At2g31690, AtDLAH/At1g30370, AtDSEL/At4g18550, AtDAD1/At2g44810), Glycine max (Glyma.08G362700, Glyma.18G299300), Ricinus communis (28424.t000001(Rc)) and Medicago truncatula (Medtr7g006400); from monocots: Oryza sativa (LOC_Os11g19340, LOC_Os11g19290, OsEG1, LOC_Os01g67450, LOC_Os02g42170), Brachypodium distachyon (Bradi4g20220, Bradi4g20230, Bradi2g57760), Zea mays (GRMZM2G117627_T0, GRMZM5G812425_T01, GRMZM2G406951_T01), Sorghum bicolor (Sobic.005G111400, Sobic.007G187000, Sobic.003G390900, Sobic.003G391000); and moss Physcomitrella patens (Phpat.022G001500). Gene IDs come from JGI website. chlo, chloroplast; mito, mitochondria; other, without a chlo/mito targeting peptide. (TIF) [file pgen.1006152.s014.tif]
